# The molecular pathogenesis of schwannomatosis, a paradigm for the co-involvement of multiple tumour suppressor genes in tumorigenesis

**DOI:** 10.1007/s00439-016-1753-8

**Published:** 2016-12-05

**Authors:** Hildegard Kehrer-Sawatzki, Said Farschtschi, Victor-Felix Mautner, David N. Cooper

**Affiliations:** 1 Institute of Human Genetics, University of Ulm, Albert-Einstein-Allee 11, 89081 Ulm, Germany; 2 Department of Neurology, University Hospital Hamburg Eppendorf, 20246 Hamburg, Germany; 3 Institute of Medical Genetics, School of Medicine, Cardiff University, Cardiff, CF14 4XN UK

## Abstract

Schwannomatosis is characterized by the predisposition to develop multiple schwannomas and, less commonly, meningiomas. Despite the clinical overlap with neurofibromatosis type 2 (NF2), schwannomatosis is not caused by germline *NF2* gene mutations. Instead, germline mutations of either the *SMARCB1* or *LZTR1* tumour suppressor genes have been identified in 86% of familial and 40% of sporadic schwannomatosis patients. In contrast to patients with rhabdoid tumours, which are due to complete loss-of-function *SMARCB1* mutations, individuals with schwannomatosis harbour predominantly hypomorphic *SMARCB1* mutations which give rise to the synthesis of mutant proteins with residual function that do not cause rhabdoid tumours. Although biallelic mutations of *SMARCB1* or *LZTR1* have been detected in the tumours of patients with schwannomatosis, the classical two-hit model of tumorigenesis is insufficient to account for schwannoma growth, since *NF2* is also frequently inactivated in these tumours. Consequently, tumorigenesis in schwannomatosis must involve the mutation of at least two different tumour suppressor genes, an occurrence frequently mediated by loss of heterozygosity of large parts of chromosome 22q harbouring not only *SMARCB1* and *LZTR1* but also *NF2*. Thus, schwannomatosis is paradigmatic for a tumour predisposition syndrome caused by the concomitant mutational inactivation of two or more tumour suppressor genes. This review provides an overview of current models of tumorigenesis and mutational patterns underlying schwannomatosis that will ultimately help to explain the complex clinical presentation of this rare disease.

**Electronic supplementary material:**

The online version of this article (doi:10.1007/s00439-016-1753-8) contains supplementary material, which is available to authorized users.

## Introduction

Schwannomatosis (MIM #162091) is a rare disorder with an estimated incidence of 1/40,000–1/70,000 (Koontz et al. 2013) that is characterized by the occurrence of multiple schwannomas and, much less commonly, meningiomas. In patients with schwannomatosis, schwannomas commonly affect the spine (74%) and peripheral nerves (89%), whereas cranial nerve schwannomas (mostly trigeminal) are uncommon (8%) (Merker et al. 2012). In one-third of patients with schwannomatosis, the tumours are anatomically limited to a single limb or several contiguous segments of the spine or one half of the body (MacCollin et al. 1996; Merker et al. 2015). The most common symptom reported by schwannomatosis patients is chronic pain which may be either local or diffuse (MacCollin et al. 2005; Merker et al. 2015).

Considerable overlap has been noted between schwannomatosis and NF2 (MIM #101000) in terms of the occurrence of the associated types of tumour, but both diseases are regarded as separate clinical entities (MacCollin et al. 1996, 2003; Evans et al. 1997; reviewed by Blakeley and Plotkin 2016). Despite this clinical overlap, there are several important differences between schwannomatosis and NF2 in relation to the frequency of specific tumour types and the occurrence of certain clinical symptoms (see Table 1 and references therein). Intradermal schwannomas, ependymomas, cataract, and retinal abnormalities are all observed in patients with NF2 but are not associated with schwannomatosis. Furthermore, bilateral vestibular schwannomas, the hallmark feature of NF2, have not been reported in patients with schwannomatosis. However, unilateral vestibular schwannomas may occur in association with schwannomatosis and hence cannot be used as an exclusion criterion to distinguish between schwannomatosis and NF2 (Smith et al. 2012a, 2015, 2016; Wu et al. 2015; Mehta et al. 2016).

**Table 1:** Clinical overlap and differences between NF2 and schwannomatosis

Clinical features	Frequency of clinical feature			
	NF2 (references)		Schwannomatosis (references)	
Bilateral vestibular schwannoma	90–95%	Evans et al. (1992), Parry et al. (1994), Mautner et al. (1996)	Absent	MacCollin et al. (1996), Merker et al. (2012)
Unilateral vestibular schwannoma	18%^a^	Evans et al. (1999)	Rare^b^	
Intracranial nonvestibular schwannoma	24–51%	Parry et al. (1994), Mautner et al. (1996), Fisher et al. (2007)	9–10%	Merker et al. (2012), Li et al. (2016)
Intracranial meningioma	45–58%	Evans et al. (1992), Parry et al. (1994), Mautner et al. (1996), Patronas et al. (2001)	5%	Merker et al. (2012)
Spinal tumour	63–90%	Parry et al. (1994), Mautner et al. (1996), Patronas et al. (2001), Dow et al. (2005), Mautner et al. (1995), Rennie et al. (2008)	74%	Merker et al. (2012)
Ependymoma	18–58%	Dow et al. (2005), Mautner et al. (1995), Rennie et al. (2008), Plotkin et al. (2011)	Absent	Gonzalvo et al. (2011), Merker et al. (2012)
Peripheral nerve schwannoma	68%	Evans et al. (1992)	89%	Merker et al. (2012)
Subcutaneous tumour^c^	43–48%	Evans et al. (1992), Mautner et al. (1997)	23%	Merker et al. (2012)
Skin plaques^d^	41–48%	Evans et al. (1992), Mautner et al. (1997)	Absent	Merker et al. (2012)
Intradermal tumour	27%	Evans et al. (1992)	Absent	MacCollin et al. (1996)
Retinal hamartoma	6–22%	Parry et al. (1994), Mautner et al. (1996), Ragge et al. (1997)	Absent	MacCollin et al. (1996)
Epiretinal membrane	12–40%	Bosch et al. (2006), Ragge et al. (1995)	Absent	MacCollin et al. (1996)
Subcapsular cataract	60–81%	Evans et al. (1992), Parry et al. (1994), Bosch et al. (2006)	Absent	MacCollin et al. (1996)

^a^Patients with unilateral vestibular schwannoma and other NF2-related tumours who fulfil the Manchester criteria (Evans et al. 2005) have a high risk of developing a contralateral tumour, especially if the patients are younger than 18 years of age at the time of diagnosis (Evans et al. 2008). Furthermore, 60% of patients with unilateral vestibular schwannomas exhibit somatic mosaicism for an *NF2* mutation (Evans et al. 2007)

^b^To date, germline *LZTR1* mutations have been identified in five patients with unilateral vestibular schwannoma and at least two nonvestibular, nonintradermal schwannomas (Smith et al. 2012a, 2015, 2016). A germline *SMARCB1* mutation has been identified in a single family with unilateral vestibular schwannoma (Wu et al. 2015). Mehta et al. (2016) have also reported a schwannomatosis patient exhibiting a unilateral vestibular schwannoma but without germline *SMARCB1* or *LZTR1* mutations

^c^Subcutaneous tumours are histologically schwannomas of peripheral nerves visible as nodular tumours

^d^Skin plaques are discrete, well-circumscribed, and slightly raised cutaneous lesions usually less than 2 cm in diameter. They are regarded as schwannomas and exhibit a rough surface often with hyperpigmentation and excessive hair

The majority of patients with schwannomatosis are sporadic, whereas 13–25% are familial cases (Evans et al. 1997; Antinheimo et al. 2000; MacCollin et al. 2005; Merker et al. 2012). A combination of linkage analysis in affected families and mutation screening of the *NF2* gene in schwannomas indicated that schwannomatosis is not due to germline mutations in the *NF2* gene (Jacoby et al. 1997; Kaufman et al. 2003; MacCollin et al. 2003). However, instead of constitutional (germline) *NF2* mutations, independent somatic mutations affecting both *NF2* alleles are frequently found in schwannomas of patients with schwannomatosis (Jacoby et al. 1997; Kaufman et al. 2003; Boyd et al. 2008; Hadfield et al. 2008; Sestini et al. 2008; Hutter et al. 2014; Paganini et al. 2015a; Piotrowski et al. 2014; Smith et al. 2015, 2016).

**Fig. 1:**
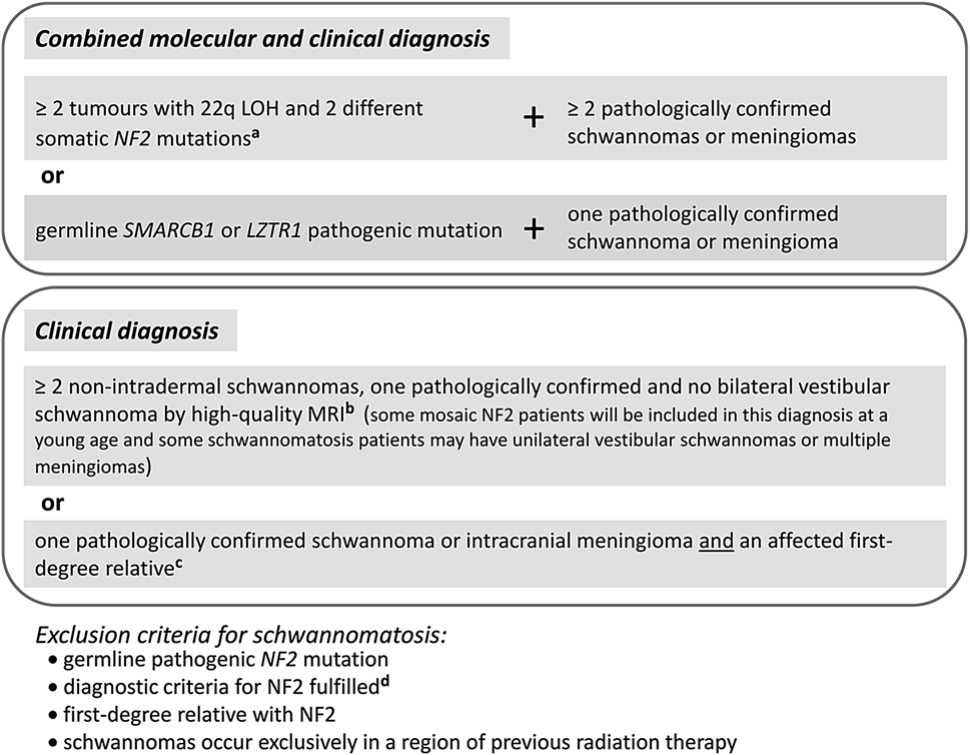
Diagnostic criteria for schwannomatosis according to Ostrow et al. (2016) and Plotkin et al. (2013) based upon the criteria formulated by MacCollin et al. (2005) which predated our ability to perform molecular testing for schwannomatosis and did not consider the possibility of multiple meningiomas. **a** According to the findings of Castellanos et al. (2015), the deletions of 22q causing the LOH in ≥ 2 tumours should have different breakpoints for these deletions to be considered as independent events. The analysis of the extent of the LOH is necessary to exclude a large 22q deletion as the first-hit mutation (that would be identical in different tumours) which would be indicative of mosaic NF2. If an identical *SMARCB1* mutation is detected in different tumours of a patient, SMARCB1-associated schwannomatosis may be diagnosed. *LZTR1*-associated schwannomatosis may be present, if an identical *LZTR1* mutation is detected in different tumours of a patient. **b** High-quality MRI should include a detailed study of the internal auditory canal with slices no more than 3 mm thick. **c** Schwannomatosis should be considered as a possible diagnosis if two or more nonintradermal tumours are present, even if none has been pathologically confirmed to be a schwannoma; the occurrence of chronic pain in association with the tumour(s) increases the likelihood of schwannomatosis (Plotkin et al. 2013). **d** Smith et al. (2016) identified five patients, with unilateral vestibular schwannomas and at least two nonvestibular, nonintradermal schwannomas, who met the diagnostic criteria for NF2 but had germline *LZTR1* mutations instead of germline *NF2* mutations. 22q LOH: loss of heterozygosity on the long arm of chromosome 22

So far, two schwannomatosis predisposition genes have been identified, *SMARCB1* and *LZTR1* (Hulsebos et al. 2007; Sestini et al. 2008; Hadfield et al. 2008; Smith et al. 2012b; Hutter et al. 2014; Piotrowski et al. 2014; Smith et al. 2015). Further schwannomatosis predisposition genes may well exist, but they still remain to be discovered. The clinical overlap between schwannomatosis and NF2 renders differential diagnosis somewhat difficult, particularly in sporadic and mosaic cases with multiple schwannomas but without bilateral vestibular schwannomas and detectable germline *NF2* gene mutations. However, comprehensive mutation testing of *LZTR1*, *SMARCB1,* and *NF2* using DNA derived from blood and different tumour samples of the patient is the method of choice to distinguish between the two conditions (Castellanos et al. 2015; Smith et al. 2016). The diagnosis of schwannomatosis is predicated upon the molecular and/or clinical diagnostic criteria according to Plotkin et al. (2013) and Ostrow et al. (2016) (Fig. 1). In what follows, we review current knowledge of the mutational patterns of the known schwannomatosis predisposing genes, models of tumorigenesis, and the genotype/phenotype relationship.

### *SMARCB1* germline mutations in patients with schwannomatosis

**Fig. 2:**
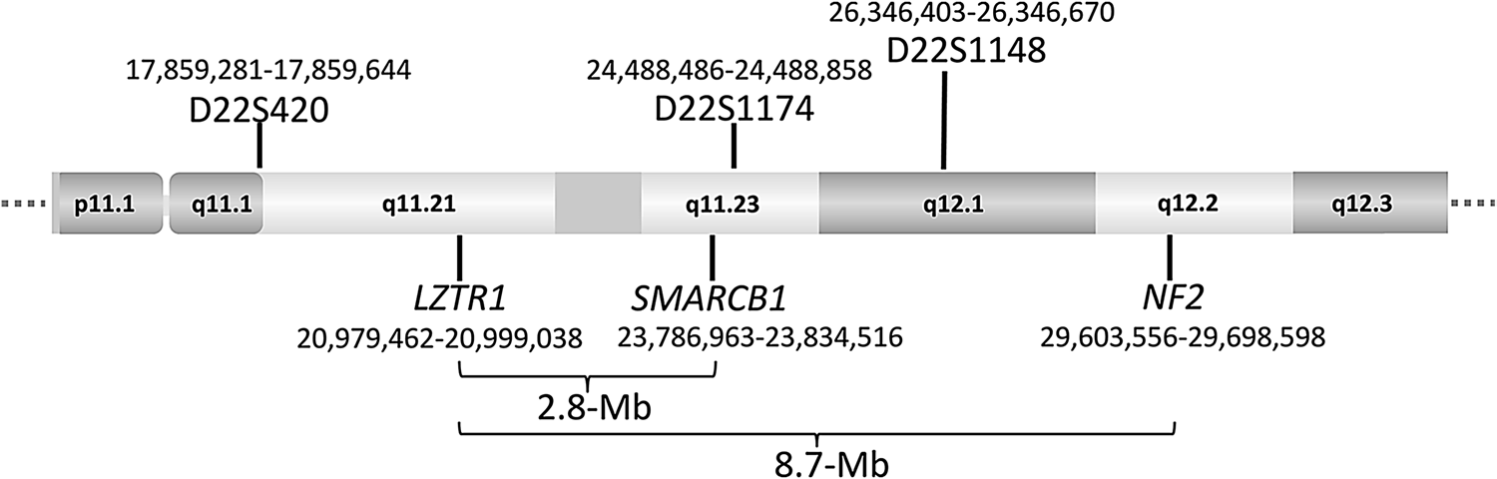
Partial ideogram of chromosome 22 indicating the location of the *LZTR1*, *SMARCB1,* and *NF2* genes and the microsatellite markers D22S420 (GenBank accession number Z23643.1), D22S1174 (GenBank acc. No. Z51327.1), and D22S1148 (GenBank acc. No. Z52647.1). The nucleotide numbering is given according to hg19. Linkage analysis provided the original evidence that the schwannomatosis predisposition genes are located within the ~8.5-Mb region between markers D22S420 and D22S1148 (MacCollin et al. 2003). The centromeric direction is on the left side, the telomeric direction is on the right side of the schema

Linkage analysis with microsatellite markers performed in families affected with schwannomatosis served to exclude the *NF2* gene as a germline-transmissible schwannomatosis predisposition gene but nevertheless suggested that such a gene could be located within an 8.48-Mb region centromeric to *NF2*, between markers D22S420 and D22S1148 in the vicinity of D22S1174 on chromosome 22 (MacCollin et al. 2003) (Fig. 2). This region includes *SMARCB1* (also termed *hSNF5* or *INI1*) which encompasses nine exons encoding a subunit of the human SWI/SNF chromatin-remodeling complex (reviewed by Kalimuthu and Chetty 2016). SMARCB1 appears not to be essential for the assembly of the remodeling complex (Doan et al. 2004) but is involved in targeting the SWI/SNF complex to gene promoters (Kuwahara et al. 2013). SMARCB1 also participates in a number of protein–protein interactions involving transcription factors, such as c-MYC and GLI1 (Cheng et al. 1999; Jagani et al. 2010; Stojanova et al. 2016). Biallelic *SMARCB1* inactivation has been detected in a multitude of different tumour types and at high frequency in rhabdoid tumours (reviewed by Roberts and Biegel, 2009; Hollmann and Hornick 2011; Masliah-Planchon et al. 2015). Using a candidate gene approach, Hulsebos et al. (2007) investigated whether *SMARCB1* might be the anticipated schwannomatosis predisposition gene. They subsequently found a germline mutation of *SMARCB1* in exon 1 (c.34C > T) that was predicted to result in premature translational termination at protein position p.Gln12* in a 22-year-old female patient and her affected father. In one schwannoma from the father, Hulsebos et al. (2007) detected an additional somatic truncating *SMARCB1* mutation (c.544C > T; p.Gln182*). In a second schwannoma from the same individual, the partial loss of the *SMARCB1* wild-type allele was observed. These findings constituted good *prima facie* evidence that *SMARCB1* functions as a tumour suppressor gene and that mutations in this gene predispose to schwannomatosis. Since this initial report, further studies have confirmed this conclusion, since germline *SMARCB1* mutations have been identified in schwannomatosis patients from different cohorts (Supp. Table S1) (Boyd et al. 2008; Hadfield et al. 2008; Sestini et al. 2008; Rousseau et al. 2011; Smith et al. 2012b, 2014; Asai et al. 2015). These studies imply that germline *SMARCB1* mutations are present in at least 48% of familial and 9.8% of sporadic schwannomatosis cases (Supp. Table S2).

#### Meningiomas in patients with schwannomatosis and germline SMARCB1 mutations

Biallelic inactivation of *SMARCB1* has been observed in both schwannomas and meningiomas of patients with schwannomatosis (Boyd et al. 2008; Sestini et al. 2008; Hadfield et al. 2008, 2010a; Smith et al. 2012b). Meningiomas occur in 5% of schwannomatosis patients (Merker et al. 2012) and appear to be located predominantly in the cerebral falx (van den Munckhof et al. 2012). Three different families have been identified in which some members harboured germline *SMARCB1* mutations and exhibited multiple schwannomas and meningiomas (Bacci et al. 2010; Christiaans et al. 2011; van den Munckhof et al. 2012; Melean et al. 2012) (Supp. Table S3). However, not all mutation carriers in these families had meningiomas, indicative of the variable expression of meningiomas in patients with germline *SMARCB1* mutations. Furthermore, *SMARCB1* germline mutations have not been found in patients with multiple meningiomas in the absence of schwannomatosis (Hadfield et al. 2010b). Multiple meningiomas are very rare and usually occur in the context of NF2 (reviewed by Smith 2015). Remarkably, germline mutations in *SMARCE1*, another component of the SWI/SNF chromatin-remodeling complex, have been identified in patients with familial multiple spinal meningiomas without NF2 (Smith et al. 2013).

#### Coffin–Siris syndrome, schwannomatosis, and SMARCB1 germline mutation

Recently, a patient with Coffin–Siris syndrome (MIM# 135900) and schwannomatosis has been reported to carry a germline missense mutation in exon 9 of *SMARCB1* (c.1121G > A; p.Arg374Gln) (Gossai et al. 2015). The patient exhibited intellectual disability, hypotonia, microcephaly, coarse face, hypoplasia of the digits, general hirsutism, multiple schwannomas, as well as bilateral cataracts and bilateral cranial nerve schwannomas which are most unusual in the context of schwannomatosis. Patients with Coffin–Siris syndrome and germline *SMARCB1* mutations have been previously reported, but none have exhibited schwannomas (Tsurusaki et al. 2012).

### Mutational pattern in *SMARCB1*-positive schwannomas as compared with rhabdoid tumours

The recognition that mutations in the *SMARCB1* gene predispose to benign schwannoma, which usually become symptomatic during adulthood, came as something of a surprise, since this gene was originally discovered in the context of its involvement in the development of atypical teratoid/rhabdoid tumours (Versteege et al. 1998; Sévenet et al. 1999a). These highly malignant tumours develop most commonly in the central nervous system in very young children who frequently die as a consequence of the malignancy before the age of 3 (Hilden et al. 2004; Lau et al. 2015). A few patients have been reported to have survived the initial tumour for up to 26 years, albeit with multiple recurrences (reviewed by Takahashi-Fujigasaki et al. 2012). Rhabdoid tumours may also develop in the kidney and less frequently, in extrarenal tissues (reviewed by Oda and Tsuneyoshi 2006), and *SMARCB1* mutations have been identified in both renal and extrarenal rhabdoid tumours (Biegel et al. 2002; Savla et al. 2000; Kordes et al. 2010). Germline mutations of *SMARCB1* occur in approximately one-third of patients with rhabdoid tumours (Biegel et al. 1999; Sévenet et al. 1999b; Bourdeaut et al. 2011; Eaton et al. 2011). Although most of the germline *SMARCB1* mutations causing rhabdoid tumours occur *de novo*, familial cases have also been reported, with several affected members harbouring constitutional *SMARCB1* mutations and malignant rhabdoid tumours but never developing schwannomas (Sévenet et al. 1999b; Taylor et al. 2000; Ammerlaan et al. 2008). This condition is known as rhabdoid tumour predisposition syndrome 1 (RTPS1: MIM#609322). In some cases, RTPS1 is caused by gonadal mosaicism for an *SMARCB1* mutation (Sévenet et al. 1999b; Bruggers et al. 2011; Lee et al. 2002; Janson et al. 2006; Eaton et al. 2011; Gigante et al. 2016). Tumorigenesis in RTPS1 is driven by somatically acquired second-hit mutations in the wild-type *SMARCB1* allele. Biallelic inactivation of *SMARCB1* through the acquisition of two somatic mutations has been observed in the rhabdoid tumours of patients without germline mutations. Hence, *SMARCB1* plays the role of a classic tumour suppressor gene in rhabdoid tumours according to the Knudson two-hit model (Versteege et al. 1998; Biegel et al. 1999; Uno et al. 2002; Jackson et al. 2009; Kordes et al. 2010; Bourdeaut et al. 2011).

In contrast to the highly malignant rhabdoid tumours, schwannomas are generally benign and very rarely transform to malignant tumours (reviewed by Carter et al. 2012). Differences in the position and type of germline *SMARCB1* mutations have been observed in patients with schwannomatosis compared to those with rhabdoid tumours. Schwannomatosis-associated *SMARCB1* mutations are preferentially located either at the 5′or 3′ end of the gene (Hulsebos et al. 2007; Hadfield et al. 2008; Rousseau et al. 2011; Smith et al. 2012b, 2014). Several recurrent *SMARCB1* mutations have been identified in patients with schwannomatosis; the most common of these is the c.*82C > T mutation located in the 3′UTR (Supp. Table S4). By contrast, intragenic germline *SMARCB1* mutations observed in patients with rhabdoid tumour tend to be located in the central part of the gene (Smith et al. 2014). In addition to this position effect, the mutational spectra differ, with *SMARCB1* mutations in schwannomatosis patients being predominantly nontruncating, including missense and splice-site mutations as well as in-frame deletions, that lead to the production of stable transcripts (Smith et al. 2012c). By contrast, almost all germline mutations of *SMARCB1* in patients with rhabdoid tumour are either protein-truncating or alternatively whole-gene or multi-exon deletions (Bourdeaut et al. 2011; Eaton et al. 2011; Smith et al. 2014; reviewed by Biegel et al. 2014). These findings are suggestive of a genotype/phenotype correlation: loss-of-function mutations occur in patients with rhabdoid tumours, whereas schwannomatosis-associated germline *SMARCB1* mutations are predominantly hypomorphic (Smith et al. 2012c, 2014).

In accord with this postulate are the findings of Hulsebos et al. (2014a). These authors analysed four schwannomatosis-associated *SMARCB1* mutations that were located in the 5′ region of the gene and were predicted to introduce a premature translational termination codon (PTC). Two of them, c.30delC (p.Phe10Leufs*6) and c.38delA (p.Lys13Serfs*3), may be predicted to cause frameshifts generating a PTC starting at nucleotide 44. The other two *SMARCB1* mutations, c.34C > T (p.Gln12*) and c.46A > T (p.Lys16*), directly generate PTCs at their respective positions. Although transcripts containing these PTCs may reasonably be expected to be degraded by nonsense-mediated mRNA decay (NMD), stable transcripts were detected by Hulsebos et al. (2014a) in schwannoma tissue obtained from two patients harbouring either c.34C > T or c.30delC. Furthermore, Western blot analysis using frozen tumour tissue from the individual with c.30delC indicated the occurrence of a shortened protein owing to downstream translational reinitiation. This finding was confirmed by transient overexpression of a c.34C > T-containing expression vector in a cell line without endogenous SMARCB1 protein and subsequent detection of the shortened SMARCB1 protein by Western blotting. Similar overexpression experiments indicated that the mutations c.30delC, c.38delA, and c.46A > T also lead to truncated SMARCB1 proteins which may well be partially functional (Hulsebos et al. 2014a).

Many of the missense and splice-site mutations as well as in-frame deletions detected in schwannomatosis patients fail to alter *SMARCB1* transcript stability, and hence, the mutant alleles are likely to encode at least partially functional proteins, as has been concluded from cyclin D1 repression assays (Smith et al. 2012c). Loss of *SMARCB1* in rhabdoid tumours leads to upregulation of cyclin D1 and cell cycle progression. A cyclin D1 repression assay has shown that mutant SMARCB1 proteins, derived from expression plasmids harbouring the same missense and splice-site mutations noted in patients with schwannomatosis, were capable of suppressing cyclin D1 activity in a similar manner to the wild-type SMARCB1 protein (Smith et al. 2012c). Taken together, these observations suggest that in schwannomatosis, germline *SMARCB1* mutations encode proteins with some residual functionality (Smith et al. 2012c; Hulsebos et al. 2014a). By contrast, *SMARCB1* mutations in rhabdoid tumours and patients with RTPS1 are almost invariably either nonsense mutations or frameshifts, even complete gene deletions, and hence are associated with a loss-of-function (Kordes et al. 2010; Bourdeaut et al. 2011; reviewed by Biegel et al. 2014). Consequently, SMARCB1 expression is completely absent in rhabdoid tumours (Hoot et al. 2004; Judkins et al. 2004; Haberler et al. 2006; Sigauke et al. 2006; Kordes et al. 2010; reviewed by Margol and Judkins 2014).

### Mosaic SMARCB1 expression in schwannomatosis-associated schwannomas

Schwannomas in patients with germline *SMARCB1* mutations have been reported to exhibit a mosaic SMARCB1 protein expression pattern by immunohistochemical staining of tumour sections with the monoclonal BAF47 antibody specific for the C-terminal part of SMARCB1 raised against amino acids 257–359 (Hulsebos et al. 2007; Patil et al. 2008; Smith et al. 2012c). This mosaic pattern results from mixed immuno-positive and -negative nuclei, consistent with the expression of SMARCB1 in a subset of tumour cells. Considerable inter- and intra-tumoral variability has been observed with regard to the number of nuclei that exhibit SMARCB1 expression (Patil et al. 2008). The mosaic expression pattern may be explicable in terms of a subset of schwannoma cells retaining the wild-type *SMARCB1* allele, thereby enabling the synthesis of sufficient SMARCB1 protein to give rise to positive immunostaining of the respective nuclei. In other types of tumour cell, however, the loss of the *SMARCB1* wild-type allele leads to complete loss of protein expression. Although this scenario may account for some schwannomas, it cannot explain the mosaic SMARCB1 immunostaining observed in the majority of these tumours. This may be concluded from the observation that the loss of the wild-type *SMARCB1* allele is readily detectable by loss of heterozygosity (LOH) analysis using polymorphic markers, suggesting that this loss affects a large proportion of tumour cells. Consequently, the mosaic SMARCB1 expression is most likely related to the hypomorphic nature of the mutations in schwannomatosis patients that encode stable mRNA transcripts giving rise to detectable amounts of SMARCB1 protein. Since, in schwannomas of patients with schwannomatosis, the wild-type *SMARCB1* allele is often lost by deletion or monosomy 22, the SMARCB1 protein detected in schwannoma cells must be encoded by the mutant allele. The inability to detect mutant proteins in all tumour cells by immunostaining is likely to be a consequence of the instability of mutant SMARCB1 proteins (Hulsebos et al. 2014a). This instability results in immunologically nonreactive SMARCB1 protein degradation products in a proportion of the schwannoma cells. Since this degradation is likely to be a random process, some cells may still express detectable amounts of SMARCB1 protein resulting in a mosaic expression pattern when analysing schwannoma tissue sections (Hulsebos et al. 2014a, 2016).

Recently, an N-terminal region of *SMARCB1* has been identified that encodes a winged helix DNA-binding domain (Allen et al. 2015). This domain is deleted from the shortened SMARCB1 proteins encoded by the transcripts harbouring exon 1 truncating mutations investigated by Hulsebos et al. (2014a). Furthermore, N-terminally located *SMARCB1* missense mutations are predicted to destabilize the encoded protein and interfere with DNA-binding (Allen et al. 2015). Despite the reduced stability and impaired DNA-binding capacity of proteins encoded by transcripts harbouring mutations located within the N-terminal half of *SMARCB1*, it would appear that these proteins retain sufficient residual function to prevent the occurrence of highly malignant rhabdoid tumours during the early childhood in patients with complete loss-of-function *SMARCB1* mutations.

### Malignancy in patients with *SMARCB1* germline mutations and schwannomatosis

Three families have been reported with germline *SMARCB1* mutation carriers presenting either with rhabdoid tumours or schwannomatosis (Swensen et al. 2009; Eaton et al. 2011; Sredni and Tomita 2015). The *SMARCB1* mutations identified in these families were predicted to introduce premature termination codons leading to the production of unstable transcripts that would then undergo nonsense-mediated mRNA decay (Supp. Table S5). Somatic mosaicism for the *SMARCB1* mutation in the family members affected only by schwannomas cannot explain the absence of rhabdoid tumours in these patients. The existence of adult mutation carriers in these families without rhabdoid tumours is intriguing and suggests that the risk of rhabdoid tumour development is time-dependent in the sense that there may be a specific developmental time window during which the progenitor cells of rhabdoid tumours are vulnerable to SMARCB1 protein loss (Boyd et al. 2008; Biegel et al. 2014). If the cells manage to progress through this critical time period without experiencing complete SMARCB1 loss, then the individual harbouring them may not develop rhabdoid tumours despite the presence of the germline *SMARCB1* mutation. This hypothesis is consistent with the peak incidence of rhabdoid tumours at 6 months of age in patients with germline *SMARCB1* mutations. Furthermore, the risk of developing a rhabdoid tumour decreases dramatically after 3 years of age (Eaton et al. 2011).

Swensen et al. (2009) investigated a four-generation family with members affected either by schwannomatosis or rhabdoid tumour (Supp. Table S5). Two schwannomas from different affected members of this family were biopsied and classified as epithelioid schwannomas. This histological subtype is very rare among schwannomas detected in patients with schwannomatosis (Hart et al. 2016). Furthermore, both schwannomas were negative for SMARCB1 immunostaining, indicative of a complete loss of the SMARCB1 protein (Swensen et al. 2009). This is in stark contrast to the schwannomatosis-associated tumours which usually exhibit mosaic SMARCB1 expression (Hulsebos et al. 2007; Patil et al. 2008; Smith et al. 2012c). Taken together, these observations suggest that schwannomas observed in this family (and probably also in other families with rhabdoid tumour and schwannomatosis) may have a different biology compared with classical schwannomatosis-associated schwannomas.

In line with this postulate are the findings of Carter et al. (2012) who reported a patient with a germline *SMARCB1* frameshift mutation in exon 3 and several schwannomas which were classified as ‘neuroblastoma-like’ (Supp. Table S5). Neuroblastoma-like schwannomas are extremely rare; only one other case of a patient possibly affected by schwannomatosis and a neuroblastoma-like schwannoma has been reported to date (Sulhyan et al. 2015). The female propositus described by Carter et al. (2012) had three children who carried the *SMARCB1* mutation and two of them suffered from rhabdoid tumour. The propositus developed an epithelioid malignant peripheral nerve sheath tumour (MPNST) with rhabdoid features arising from a preexisting schwannoma. However, the malignant transformation of schwannomas is extremely rare and has been observed in only a few cases (Woodruff et al. 1994; Nayler et al. 1996; McMenamin and Fletcher 2001).

The above notwithstanding, malignant schwannomas were reported in two patients with familial schwannomatosis (Gonzalvo et al. 2011). However, it is unknown whether they carried *SMARCB1* germline mutations. Furthermore, MPNSTs were also noted in two members of a family with schwannomatosis and two co-occurring *SMARCB1* alterations, a missense mutation in exon 7 (c.864C > G; p.Asn288Lys) and a splice-site mutation located 12-bp upstream of exon 9 (c.1032-12C > G) which is predicted to lead to the insertion of 11 bp of intronic sequence in the mutant transcript (Hadfield et al. 2008; Evans et al. 2012; Smith et al. 2012b). This insertion would be predicted to introduce a frameshift that would result in the introduction of a novel stop codon (p. Arg373fsX379*). These findings raised some concern about an increased MPNST risk in schwannomatosis patients, but neither MPNSTs nor rhabdoid tumours were observed in a cohort of 87 patients with schwannomatosis, including 11 patients from seven families (Merker et al. 2012). However, it is not known how many of the patients investigated by Merker et al. (2012) carried *SMARCB1* germline mutations. Further studies are necessary to assess the MPNST risk for patients with *SMARCB1* mutation-positive schwannomatosis.

A risk of malignancy may exist in those schwannomatosis patients with *SMARCB1* germline mutations that are less likely to be hypomorphic. Paganini et al. (2015a) reported a patient with schwannomatosis and a uterine leiomyosarcoma. The patient had a germline *SMARCB1* mutation c.1118G > A involving the last nucleotide of exon 8, disrupting the donor splice site of intron 8. RNA analysis indicated that this mutation leads to the insertion of intron 8 sequences in the transcript that would be predicted to result in a frameshift. The mutant RNA has been shown to encode a C-terminally elongated protein exhibiting a different amino acid sequence and a new stop codon after an additional 48 amino acids. LOH analysis indicated that the mutant *SMARCB1* allele was retained in two schwannomas and the leiomyosarcoma of the patient, whereas the wild-type allele was lost, indicative of biallelic *SMARCB1* inactivation in both types of tumour (Paganini et al. 2015a). Substantiating concern about an increased risk of malignancy in a subset of patients with *SMARCB1*-positive schwannomatosis, Hulsebos et al. (2016) reported a type 1 papillary renal cell carcinoma (pRCC1) in a schwannomatosis patient with a germline duplication of *SMARCB1* exon 7 (c.796-2246_9861 +5250dup7686) resulting in a premature stop codon (p.Glu330*). The chromosome 22 carrying the mutant *SMARCB1* allele was retained in the pRCC1 and in schwannomas from this patient, whereas the wild-type *SMARCB1* allele and one *NF2* copy were lost by deletion. Immunohistochemical staining revealed the complete loss of SMARCB1 expression in the pRCC1 (Hulsebos et al. 2016) as observed in malignant rhabdoid tumours with biallelic *SMARCB1* inactivation.

The leiomyosarcoma and the pRCC1 observed in two patients with *SMARCB1* mutation-positive schwannomatosis indicate that malignancy may, indeed, occur in a subset of patients. However, the risk is probably dependent upon the *SMARCB1* mutation type and may be decreased in patients with hypomorphic *SMARCB1* mutations.

### Models of tumorigenesis in *SMARCB1*-associated schwannomatosis

**Fig. 3:**
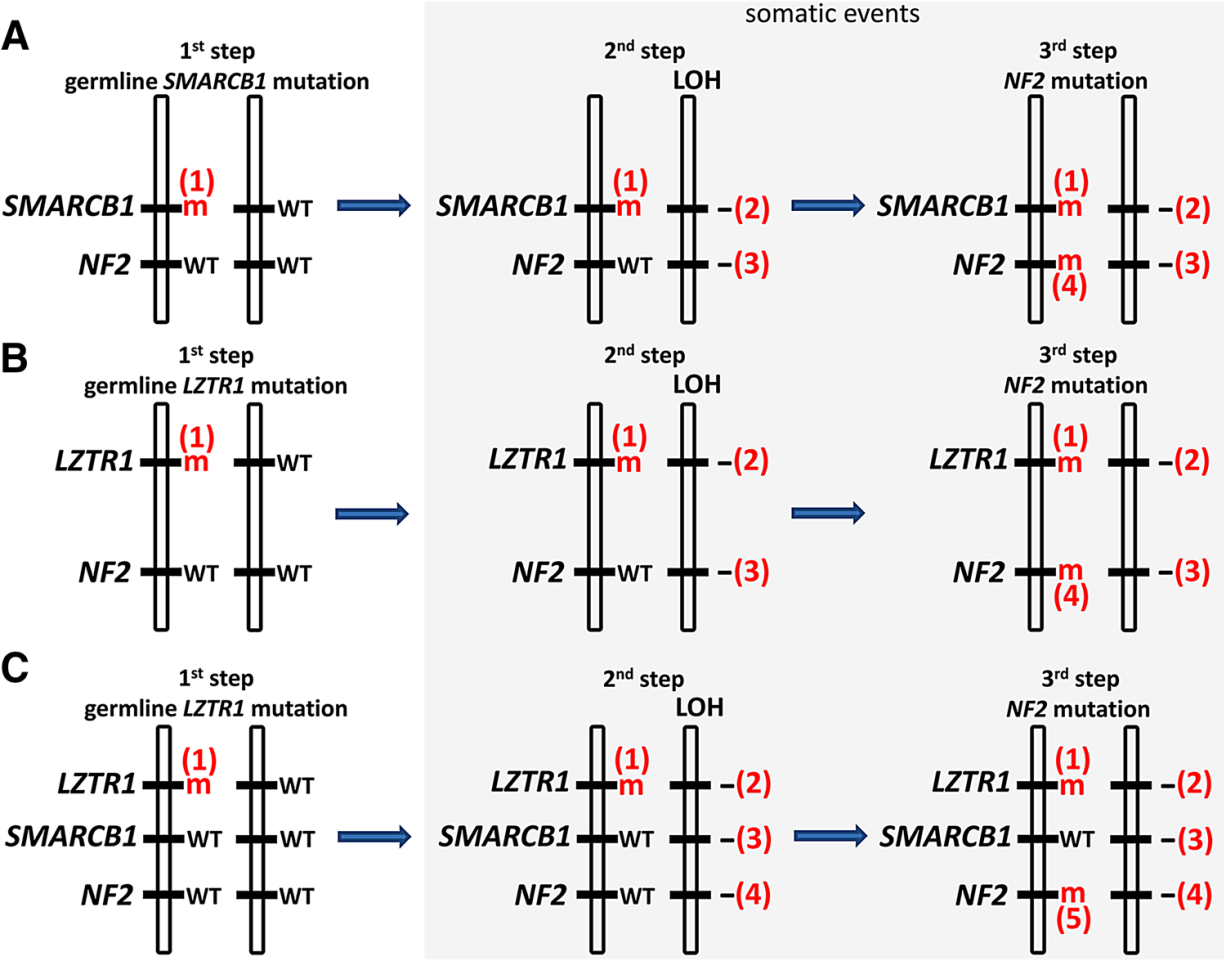
Models of tumorigenesis in schwannomatosis. **a** Four-hit/3-step model in patients with an heterozygous germline *SMARCB1* mutation (first hit and step). The second step includes loss of heterozygosity (LOH) of 22q which removes the wild-type *SMARCB1* allele and one of the two *NF2* alleles. The third step is the somatic mutation of the other *NF2* allele located on the chromosome harbouring the germline *SMARCB1* mutation. **b** Four-hit/3-step model of tumorigenesis in patients with an heterozygous germline *LZTR1* mutation (first hit and step). **c** Five-hit/3-step model of tumorigenesis in schwannomatosis. The LOH event which removes one wild-type *LZTR1* allele and one copy of *NF2* automatically leads to the loss of one *SMARCB1* allele, which represents the fifth mutational hit

The classical two-hit model of tumorigenesis (Knudson 1971) does not seem to pertain in the tumours of patients with *SMARCB1* germline mutations, at least in the sense that this model would require biallelic *SMARCB1* inactivation to be sufficient for tumour initiation or growth. This is concluded from the observation of frequent somatic, tumour-specific *NF2* mutations, and the loss of the second *NF2* allele in schwannomas from patients with germline *SMARCB1* mutations (Boyd et al. 2008; Sestini et al. 2008; Hadfield et al. 2008, 2010a). These findings hint at a significantly higher level of complexity than that required by the basic Knudson model. The loss of the second *NF2* allele is frequently caused by the complete loss of chromosome 22 or large portions of the long arm of chromosome 22 (22q), including the second (wild-type) allele of *SMARCB1* (Hadfield et al. 2010a). This pattern of mutational events points to a 4-hit/3-step model of tumorigenesis in patients with *SMARCB1*-positive schwannomatosis (Fig. 3a): The first hit (and step) represents the germline *SMARCB1* mutation, whereas the second step involves LOH of 22q that removes the wild-type *SMARCB1* allele and one of the two *NF2* alleles. The third step is the somatic mutation of the remaining *NF2* allele located on the chromosome harbouring the germline *SMARCB1* mutation which is retained in the tumour (Boyd et al. 2008; Hadfield et al. 2008; Sestini et al. 2008). This 4-hit/3-step model of tumorigenesis probably also holds true for other benign tumours observed in patients with *SMARCB1*-positive schwannomatosis, since biallelic inactivation of *SMARCB1* and *NF2* has been observed in a meningioma and leiomyoma of patients with germline *SMARCB1* mutations (van den Munckhof et al. 2012; Hulsebos et al. 2014b).

LOH of 22q in tumours from patients with germline *SMARCB1* mutations does not seem to be mediated by mitotic recombination, a mechanism frequently causing LOH of other tumour suppressor genes (Makishima and Maciejewski 2011; Garcia-Linares et al. 2011; Stewart et al. 2012). In eight tumours from patients with germline *SMARCB1* mutations, LOH of 22q was found to be caused exclusively by whole chromosome loss or large deletions within 22q, but not by mitotic recombination (Hadfield et al. 2010a). Copy-number neutral LOH of the *NF2* locus indicative of mitotic recombination was also not observed in 17 schwannomas from patients with germline *SMARCB1* mutations analysed by Piotrowski et al. (2014). By contrast, mitotic recombination accounts for 19% of the LOH events in NF2-associated schwannomas (14 of 72 schwannomas analysed) and 23% (5/22) of schwannomas from schwannomatosis patients without germline *SMARCB1* mutations (Hadfield et al. 2010a). The observation that LOH in schwannomas of patients with *SMARCB1* germline mutations is not mediated by mitotic recombination supports the 4-hit/3-step model of tumorigenesis from the standpoint of maximum parsimony (Fig. 3a). Although mitotic recombination would lead to biallelic inactivation of *SMARCB1* by reduplication of the mutant *SMARCB1* allele, the wild-type *NF2* allele would be preserved. Two additional independent mutational events would then be required to inactivate both *NF2* alleles. Hence, mitotic recombination would make four steps of mutation necessary to inactivate both alleles of *SMARCB1* and *NF2* instead of three as suggested by the model of tumorigenesis for *SMARCB1*-positive schwannomatosis depicted in Fig. 3a.

Although the 4-hit/3-step model accounts for the majority of the tumours in the cases of *SMARCB1* mutation-positive schwannomatosis, biallelic inactivation of the *NF2* gene is not observed in all schwannomas (Boyd et al. 2008; Hadfield et al. 2008, 2010a; Sestini et al. 2008; Hulsebos et al. 2007, 2016). It is estimated that at least 19% of schwannomas of patients with germline *SMARCB1* mutations exhibit only mono-allelic *NF2* inactivation (Supp. Table S6) (Boyd et al. 2008; Hadfield et al. 2008, 2010a; Sestini et al. 2008). In this subgroup of tumours, the inactivation of only one *NF2* allele may be sufficient to promote the proliferation of the cells with biallelic *SMARCB1* inactivation. Since the LOH events in *SMARCB1* mutation-positive schwannoma usually include large parts of 22q (Hadfield et al. 2010a), other tumour suppressor genes located on 22q will also be haploinsufficient and this may contribute to schwannoma growth. One of these suspected tumour suppressor genes is likely to be *LZTR1*.

## Germline mutations in *LZTR1*

Germline *SMARCB1* mutations account for 48% of familial and 9.8% of sporadic schwannomatosis cases (Supp. Table S2) indicative of probable locus heterogeneity and the existence of additional schwannomatosis predisposition genes. Piotrowski et al. (2014) analysed 3.72 Mb of highly conserved DNA sequence within different regions of 22q, including parts of the previously defined linkage interval postulated to harbour schwannomatosis predisposition genes (MacCollin et al. 2003) and the region of the *CABIN1* gene which has been suggested to be important in schwannomatosis (Buckley et al. 2005). The 3.72 Mb of highly conserved sequence were analysed by targeted next-generation sequencing which indicated germline mutations within in the *LZTR1* (leucine zipper-like transcriptional regulator 1) gene in patients with schwannomatosis but lacking *SMARCB1* mutations (Piotrowski et al. 2014). *LZTR1* is located 2.8 Mb centromeric to *SMARCB1* and 8.7 Mb centromeric to *NF2* (Fig. 2), and is included in the 1.5–3-Mb region which is deleted in individuals with the DiGeorge syndrome. The protein encoded by *LZTR1* contains Kelch- and BTB/POZ-domains and localizes to the Golgi complex (Nacak et al. 2006). It has also been shown to be an adaptor of the cullin 3-containing E3 ubiquitin ligase complex (Frattini et al. 2013). Somatic mutations in *LZTR1* occur in 22% of glioblastomas (Frattini et al. 2013) and in several other cancers according to the Catalogue of Somatic Mutations in Cancer (COSMIC) database (Forbes et al. 2008). The biallelic inactivation of *LZTR1* in glioblastomas, and the observation that *LZTR1* inactivation drives self-renewal and growth of glioma spheres, are indicative of *LZTR1* acting as a classic tumour suppressor gene (Frattini et al. 2013).

Piotrowski et al. (2014) identified *LZTR1* germline mutations in 16 of 20 unrelated schwannomatosis patients (80%). Among these, 20 patients were 6 familial schwannomatosis cases and *LZTR1* mutations were found in all of them. Eleven of the twenty patients analysed were confirmed sporadic cases and eight of these (73%) had *LZTR1* mutations (Piotrowski et al. 2014). Such a high rate of *LZTR1* mutation in *SMARCB1* mutation-negative schwannomatosis patients was not, however, observed in subsequent studies (Hutter et al. 2014; Paganini et al. 2015b; Smith et al. 2015). In these latter studies, only 22–30% of sporadic cases and 38% of familial cases exhibited *LZTR1* mutations (Supp. Table S7). This difference in the proportion of *LZTR1* mutation-positive schwannomatosis cases detected may be due to the adoption of different selection criteria for the patients being analysed. Piotrowski et al. (2014) exclusively studied patients with a molecularly confirmed diagnosis of schwannomatosis. This implies that two different somatic *NF2* mutations were identified in two tumours of a given patient as well as the loss of chromosome 22q encompassing the wild-type *NF2* allele. By contrast, the studies with lower *LZTR1* mutation detection rates mostly focussed upon patients diagnosed with schwannomatosis on the basis of clinical criteria according to Plotkin et al. (2013) and who were negative for *SMARCB1* mutations. Consequently, the studies reporting lower *LZTR1* mutation detection rates included a more heterogeneous group of patients whose schwannomas may not have been characterized by biallelic *NF2* inactivation. Nevertheless, these studies serve to confirm that *LZTR1* is a major schwannomatosis predisposition gene.

Schwannomatosis-associated *LZTR1* mutations were found in nearly all exons; thus, no positional preference of mutations was observed (Hutter et al. 2014; Piotrowski et al. 2014; Paganini et al. 2015a; Smith et al. 2015). In contrast to this situation, *SMARCB1* mutations causing schwannomatosis are predominantly located at the 5′ or 3′ end of the gene (Smith et al. 2014). Of the 59 *LZTR1* mutations identified to date, at least 28 (47%) were protein-truncating, whilst all of the 23 missense mutations were predicted to be deleterious (Supp. Table S8). These findings suggest that the majority of schwannomatosis-associated *LZTR1* mutations are not hypomorphic. Somatic loss of the wild-type *LZTR1* allele is predicted to lead to biallelic loss of *LZTR1* in tumours of germline mutation carriers. However, the type of the *LZTR1* mutation would appear to influence LZTR1 protein expression in tumours. Schwannomas from patients with nonsense or frameshift *LZTR1* mutations do not exhibit LZTR1 protein immunostaining, whereas patients carrying splicing or missense variants showed reduced immunostaining (Paganini et al. 2015b). By contrast, schwannomas from *LZTR1*-unrelated schwannomatosis patients, as well as patients with NF2, showed diffuse but positive LZTR1 immunostaining. Whether this variability has any effect on schwannoma growth or location is unclear but clearly warrants further investigation.

### *LZTR1* mutations in unaffected carriers

Ten unrelated and clinically unaffected *LZTR1* mutation carriers have been identified who had relatives harbouring the same mutation and were affected by schwannomatosis (Supp. Table S8). Eight of these ten apparently nonpenetrant *LZTR1* mutations were protein-truncating and highly likely to be deleterious. Even though not all clinically unaffected *LZTR1* mutation carriers have been investigated by MRI [precluding the unambiguous exclusion of the occurrence of minor lesions, such as intrafascicular microlesions described by Farschtschi et al. (2016)], many of these individuals are of advanced age and some clinical symptoms should already have become apparent. The reason for the incomplete penetrance of these *LZTR1* mutations is currently unclear, but it may be that somatic mosaicism for the *LZTR1* mutations accounts for the absence of clinical symptoms in the unaffected mutation carriers who passed on the mutations to the affected family members. Although the respective *LZTR1* mutations were detected in high proportions of peripheral blood cells of the unaffected family members, this should not be held to imply that numerous Schwann cell progenitor cells also harbour the mutation. We postulate that the *LZTR1* mutations in the unaffected probands are of post-zygotic origin and are present in a minority of Schwann cell precursors. The haematopoietic progenitor cells carrying the *LZTR1* mutation may possess a selective growth advantage, giving rise to a high proportion of mutation-positive blood cells. This situation would be reminiscent of patients with neurofibromatosis type 1 (NF1) and somatic mosaicism for a large deletion in the *NF1* gene region spanning 1.2 Mb. The patients have a high proportion of blood cells carrying the *NF1* deletion (>90%); a much lower proportion of cells with the *NF1* deletion has, however, been detected in the fibroblasts and urine of these patients (Kehrer-Sawatzki and Cooper, 2008; Roehl et al. 2012). Somatic mosaicism due to a post-zygotic mutation and selective growth advantage of *LZTR1* haploinsufficient blood cells may, therefore, be responsible for the apparently incomplete penetrance of some *LZTR1* mutations. However, it is possible that the *LZTR1* mutation in the unaffected family members is, indeed, constitutional and that the lack of clinical symptoms manifested by these *LZTR1* mutation carriers may be caused by as yet unidentified modifying genes.

## *LZTR1* germline mutations and associated tumour spectrum

So far, patients with schwannomatosis and *LZTR1* germline mutations have not been reported to exhibit meningiomas, in contrast to patients with *SMARCB1* germline mutations (Hutter et al. 2014; Piotrowski et al. 2015; Paganini et al. 2015b; Smith et al. 2015, 2016). These findings suggest that *LZTR1* mutations do not predispose to meningiomas. However, unilateral vestibular schwannomas can occur in patients with *LZTR1* mutation-positive schwannomatosis. Recently, Smith et al. (2016) demonstrated that 5 of 70 patients (7%) presenting with a unilateral vestibular schwannoma and at least two nonintradermal, nonvestibular schwannomas have germline *LZTR1* mutations; hence, these individuals have schwannomatosis rather than NF2 even, although they would fulfil current diagnostic criteria for NF2. These findings further evidence the overlap between schwannomatosis and NF2.

In contrast to *LZTR1*-associated schwannomatosis, *SMARCB1* mutations are probably less likely to predispose to unilateral vestibular schwannomas. So far, only one solitary case of a unilateral vestibular schwannoma in a putative S*MARCB1*-associated schwannomatosis family has been reported (Wu et al. 2015). However, this report remains inconclusive, since, although the *SMARCB1* mutation was identified in a schwannoma of family member with a unilateral schwannoma, this mutation was not confirmed to be present in the germline of this patient or in any other affected family member. Further studies are, therefore, needed to investigate a possible association between unilateral vestibular schwannomas and germline *SMARCB1* mutations.

## Segmental schwannomatosis

In approximately one-third of schwannomatosis patients, tumours are anatomically restricted to a single limb or a few adjacent spinal segments, thereby manifesting what appears to be a segmental form of the disease (MacCollin et al. 2005; Merker et al. 2012). Two patients have been identified with clinically symptomatic schwannomas restricted to one limb, suggestive of somatic mosaicism; however, germline *LZTR1* mutations were identified in these patients (Farschtschi et al. 2016). Furthermore, microstructural magnetic resonance neurography indicated the presence of small peripheral nerve lesions (‘microlesions’) in clinically unaffected limbs of these two patients. Three additional patients were included in this study and these individuals also exhibited symptomatic schwannomas that were restricted to one limb and fascicular microlesions along the nerves of other extremities. Germline mutations in *LZTR1*, *SMARCB1,* and *NF2* were not observed in these patients. However, the analysis of a schwannoma from each of these patients indicated a somatic *NF2* gene mutation which is not unusual, since somatic *NF2* mutations are frequently observed in schwannomas. The occurrence of asymptomatic peripheral nerve microlesions in multiple body parts in these five patients with segmental occurrence of clinically symptomatic schwannomas suggests that the disease manifestation is more widespread and not simply restricted to a single body segment (Farschtschi et al. 2016).

## Models of tumorigenesis in *LZTR1* mutation-positive schwannomatosis

In most schwannomas from patients harbouring *LZTR1* germline mutations, the chromosome 22 is retained that harbours the germline *LZTR1* mutation and an *NF2* allele with a somatically acquired tumour-specific mutation. The other copy of chromosome 22 is lost or partially deleted, including the wild-type alleles of *LZTR1* and *NF2* (Piotrowski et al. 2014; Paganini et al. 2015b; Smith et al. 2015) (Fig. 3b). Consequently, the mutational mechanism would appear to be similar to the 4-hit/3-step model of tumorigenesis observed in schwannomas of patients with germline *SMARCB1* mutations (Fig. 3a). However, since *SMARCB1* is located between *LZTR1* and *NF2*, both of which are lost by deletion or monosomy 22q, it is inevitable that one of the two *SMARCB1* alleles is also lost. Hence, in practice, tumorigenesis in *LZTR1*-associated schwannomatosis should follow a 5-hit/3-step model (Fig. 3c). Moreover, schwannomas of patients with germline *SMARCB1* mutations may follow the 5-hit/3-step model if the chromosomal region on chromosome 22 exhibiting LOH also includes *LZTR1*. Since *LZTR1* is located proximal to *SMARCB1* and *NF2*, the loss of one *LZTR1* allele is dependent upon the extent of the region showing LOH. It is, however, unknown if the loss of one *LZTR1* allele contributes to tumour growth. A mutation of the second *LZTR1* allele would be necessary for the biallelic inactivation of this tumour suppressor gene. It remains to be investigated if schwannomas following either the 4-hit or the 5-hit model of tumorigenesis would exhibit differences in growth rate, proliferation index, or location.

The 3-step model of tumorigenesis appears to apply to the majority of *LZTR1* mutation-positive schwannomas. However, not all schwannomas from patients with *LZTR1* germline mutations exhibit this pattern of mutational events similar to that observed in some schwannomas of patients with germline *SMARCB1* mutations. Instead of the biallelic *NF2* inactivation implied by this model, mono-allelic *NF2* inactivation was observed in 10 of the 28 (37%) schwannomas from patients with germline *LZTR1* mutations analysed by Paganini et al. (2015b). In eight of these ten schwannomas, 22q LOH was detected, but an intragenic *NF2* gene mutation could not be found, whereas two of ten schwannomas harboured an intragenic *NF2* mutation, while heterozygosity of 22q markers was retained (Paganini et al. 2015b). Mono-allelic *NF2* inactivation was detected in 3 of 11 (27%) schwannomas from *LZTR1* mutation-positive patients studied by Smith et al. (2015). However, it cannot be excluded that biallelic *NF2* inactivation was not detected in these studies owing to the limited resolution of the methodology employed. Further analysis would be necessary to establish whether alternative mechanisms, such as epigenetic silencing of *NF2* gene expression, could be involved in the biallelic *NF2* inactivation in these tumours. Indeed, hypermethylation of the *NF2* promoter region has been shown to be frequent in schwannomas and could, therefore, represent an alternative mechanism of *NF2* inactivation (Kino et al. 2001; Gonzalez-Gomez et al. 2003).

## Predisposition to bilateral vestibular schwannomas in patients with NF2 but not schwannomatosis

*NF2* mutations are detected in schwannomas irrespective of whether they occur sporadically or in the context of schwannomatosis or NF2 (Jacoby et al. 1996; Mohyuddin et al. 2002; Hadfield et al. 2010a). However, the timing of origin of the underlying mutations is different which may well impact upon the spectrum of tumours observed, in particular bilateral vestibular schwannomas (BVS). In patients with NF2, the germline *NF2* gene mutation is clearly going to be present during the early embryonic development and subsets of Schwann cell precursors may be especially vulnerable to *NF2* haploinsufficiency during a specific developmental time period. This could account for the high frequency (>90%) of BVS in patients with germline *NF2* mutations. By contrast, in patients with sporadically occurring schwannomas and in patients with schwannomatosis, the somatic *NF2* mutations occur rather later during development and neither group of patients exhibits BVS. Furthermore, the histology and growth pattern of vestibular schwannomas (VS) in NF2 are different from that of sporadic VS. VS in NF2 are multifocal, appearing “like a bunch of grapes” around the vestibular nerve (Stivaros et al. 2015). Remarkably, these multifocal tumours exhibit the same first-hit *NF2* mutation but different, foci-specific second-hit *NF2* mutations, indicative of the polyclonality of these tumours (Mohyuddin et al. 2002; Dewan et al. 2015). NF2-associated VS grow at multiple sites along the eighth cranial nerve and these tumour foci later merge into one tumour mass with a multi-lobulated histological pattern (Stivaros et al. 2015). By contrast, sporadic VS are usually single tumours arising from the vestibular nerve at the porus acusticus. NF2-associated VS are often more aggressive and difficult to treat than sporadic VS because of their growth pattern and the involvement of nerves. To date, seven cases of unilateral VS have been reported in patients with schwannomatosis (Smith et al. 2012a, 2015, 2016; Wu et al. 2015; Mehta et al. 2016), but neither their histology nor their growth pattern has been described.

## Involvement of *SMARCB1* in NF2-associated schwannomas

The main focus of this review is schwannomatosis, but the considerable clinical overlap between NF2 and schwannomatosis, as well as the shared mutational mechanisms (such as LOH of 22q), render it likely that a similar set of genes is involved in both conditions.

NF2-associated and sporadically occurring schwannomas are known to be caused by biallelic inactivation of the *NF2* gene (Stemmer-Rachamimov et al. 1997; reviewed by Evans 2009). LOH of large parts of 22q is observed in 67% of NF2-associated and 56% of sporadic schwannomas (Hadfield et al. 2010a). However, copy-number neutral LOH mediated by mitotic recombination is relatively frequent, observed in 19% of NF2-associated schwannomas and in 6% of sporadic schwannomas. Nevertheless, LOH causing copy-number loss in 22q represents the most frequent second-hit mutation observed in ~50% of all NF2-associated schwannomas (Hadfield et al. 2010a) and 69% of sporadic schwannomas (Agnihotri et al. 2016). Since the LOH frequently involves large portions of chromosome 22q (Mantripragada et al. 2003; Warren et al. 2003), it is likely to be associated with the concomitant loss of several tumour suppressor genes. It has been suggested that more than two mutations are necessary for vestibular schwannoma development in NF2 patients (Woods et al. 2003). Indeed, LOH involving large parts of 22q is a key mutational step to mediate the concurrent loss of *NF2*, *SMARCB1* and eventually also *LZTR1*. It should be noted that 83% of all NF2-associated schwannomas exhibit a mosaic SMARCB1 protein expression pattern resulting from intermixed tumour cells with and without SMARCB1 expression (Patil et al. 2008). This mosaic pattern has also been observed in 93% of schwannomas from patients with familial schwannomatosis and 55% of schwannomas from patients with sporadic schwannomatosis (Patil et al. 2008). However, only 5% of sporadically occurring schwannomas in patients without NF2 or schwannomatosis exhibit mosaic SMARCB1 expression. These findings suggest the frequent involvement of *SMARCB1* in the pathogenesis of NF2 and schwannomatosis-associated tumours but not in sporadic schwannomas. Remarkably, the loss of LZTR1 immunostaining was not observed in seven vestibular schwannomas from seven unrelated NF2 patients (Paganini et al. 2015b).

## Somatic mosaicism in schwannomatosis and unknown schwannomatosis predisposition genes

**Fig. 4:**
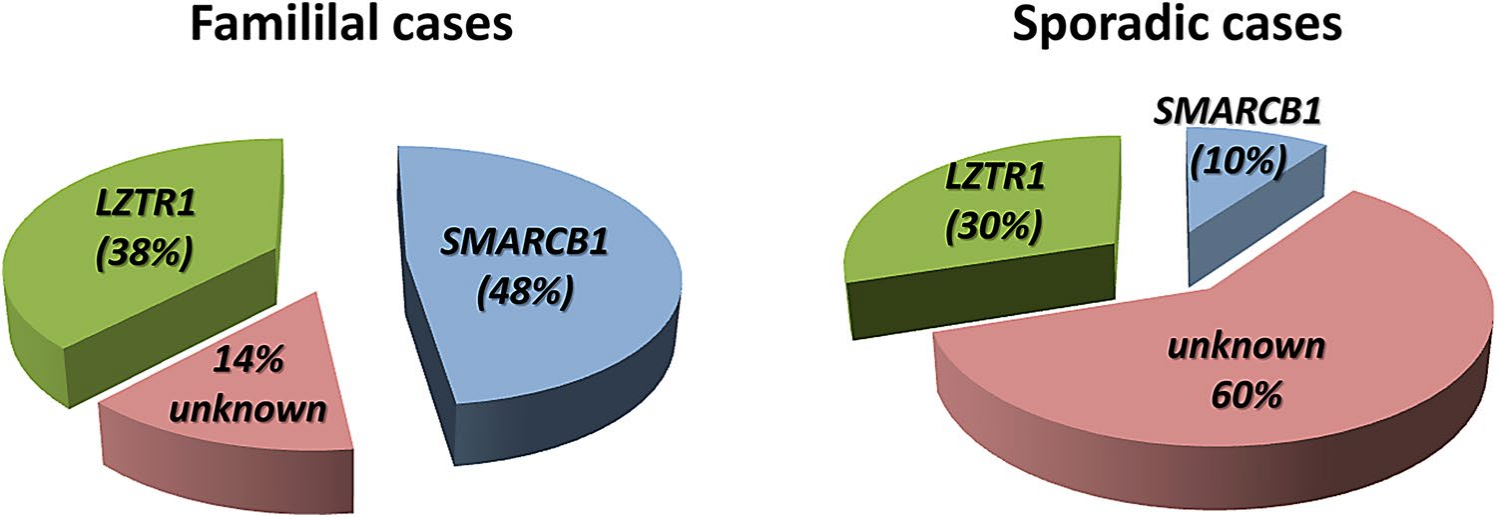
Estimation of the relative proportions of familial and sporadic patients with germline mutations in *LZTR1* or *SMARCB1* among patients who fulfil the clinical diagnostic criteria for schwannomatosis. These estimates are derived from studies of individuals diagnosed with schwannomatosis according to clinical diagnostic criteria without preselection for those patients who have been shown to harbour different somatic *NF2* gene mutations in at least two different schwannomas (Boyd et al. 2008; Hadfield et al. 2008; Sestini et al. 2008; Rousseau et al. 2011; Hutter et al. 2014; Smith et al. 2012b, 2014, 2015)

Mutation screening performed with blood-derived DNA from patients with schwannomatosis suggests that 38% of familial cases are caused by *LZTR1* mutations and 48% by mutations in *SMARCB1.* Thus, in 14% of familial schwannomatosis cases, predisposing germline mutations have not been identified as yet. In sporadic schwannomatosis, 30% of the cases are caused by germline *LZTR1* mutations and 10% by germline *SMARCB1* mutations (Supp. Tables S2 and S7) (Boyd et al. 2008; Hadfield et al. 2008; Sestini et al. 2008; Rousseau et al. 2011; Smith et al. 2012b, 2014, 2015; Hutter et al. 2014; Paganini et al. 2015b). According to these assessments, the causative mutational events remain unknown in 60% of all sporadic schwannomatosis patients (Fig. 4). It is possible that a proportion of patients without intragenic germline *SMARCB1* or *LZTR1* mutations may harbour mutations in remote-acting regulatory regions or possess epimutations that would silence these genes, but this has not so far been investigated. Alternatively, somatic mosaicism for *SMARCB1* or *LZTR1* mutations could be responsible for the relatively high proportion of sporadic schwannomatosis patients without detectable germline *SMARCB1* and *LZTR1* mutations. However, no detectable mosaicism was found in six patients without *SMARCB1* and *LZTR1* mutations in their blood, as determined by analysing multiple schwannomas from these patients. In these tumours, neither *SMARCB1* nor *LZTR1* mutations were detected. Instead, tumour-specific somatic *NF2* mutations were identified which are known to be frequent in schwannomatosis (Paganini et al. 2015b; Smith et al. 2015). Mosaicism for somatic *LZTR1* or *SMARCB1* mutations in patients with schwannomatosis has not so far been reported and only one case of germline (gonadal) mosaicism for a *SMARCB1* mutation has been identified (Hulsebos et al. 2010). Taken together, we surmise that it is unlikely that unidentified somatic mosaicism for *SMARCB1* or *LZTR1* mutations would account for the high number of sporadic schwannomatosis cases without identified mutations.

On the other hand, somatic mosaicism for an *NF2* gene mutation may be much more frequent in patients considered to have schwannomatosis, but who do not carry germline *SMARCB1* or *LZTR1* mutations as suggested by Widemann et al. (2014). Indeed, some NF2 patients with somatic mosaicism for an *NF2* gene mutation fulfil the diagnostic criteria for schwannomatosis (Plotkin et al. 2013). Further, somatic mosaicism in NF2 is not rare, since it is detected in 33% of sporadic NF2 cases with bilateral vestibular schwannomas and in up to 60% of patients with unilateral vestibular schwannoma (Moyhuddin et al. 2003; Evans et al. 2007). In patients with mosaic NF2, the ‘first-hit’ *NF2* gene mutation is often present at a low level in blood cells and is sometimes only detectable in tumour tissue but not in blood cells (Evans et al. 1998, 2007; Kluwe and Mautner 1998; Kluwe et al. 2003; Paganini et al. 2014; Spyra et al. 2015; Smith et al. 2016). To distinguish between mosaic NF2 and schwannomatosis, it is necessary to perform *NF2* mutation testing in more than one tumour from a given patient who does not carry a germline mutation in *SMARCB1* or *LZTR1* as determined by blood cell analysis. Mosaic NF2 would be confirmed if the same *NF2* mutation (first-hit) is observed in two independent tumours from a given patient in addition to different tumour-specific (second-hit) mutations of the other *NF2* allele. This strategy has been successfully pursued by Castellanos et al. (2015) who investigated a female patient with several painful schwannomas confined to one limb. In the schwannomas, but not the blood cells of this patient, Castellanos et al. detected a large deletion encompassing not only the *NF2* gene but also large parts of chromosome 22 telomeric to *NF2*. Neither *SMARCB1* nor *LZTR1* was included within the bounds of this deletion, since these genes are located centromeric to *NF2*. Importantly, the deletion exhibited the same breakpoint in both schwannomas from this patient, indicating that the deletion represented the first-hit mutation. By contrast, two different intragenic *NF2* gene mutations were identified which were specific to each tumour and hence represented the second-hit mutations. Consequently, the genetic diagnosis of this patient was mosaic NF2 rather than schwannomatosis. The study of Castellanos et al. (2015) demonstrates how important it is to distinguish between first-hit and second-hit mutations by the meticulous analysis of deletion breakpoints and extent of LOH to arrive at a precise diagnosis by means of molecular genetic testing.

Even if it is assumed that a certain proportion of unexplained schwannomatosis cases are caused by somatic mosaicism for an *NF2* gene mutation, a subset of these unexplained cases may well be caused by mutations in a gene or genes that still remain to be identified. This subset should comprise at least 27% of all unexplained sporadic schwannomatosis cases, as concluded from the analysis of Piotrowski et al. (2014). To minimize the confounding influence of patients with somatic mosaicism for *NF2* gene mutations among patients with suspected schwannomatosis, Piotrowski et al. (2014) included in their analysis of *SMARCB1* mutation-negative patients only those individuals who exhibited different somatic *NF2* gene mutations and LOH of chromosome 22q in at least two different tumours analysed. They were able to detect *LZTR1* mutations in all six familial schwannomatosis cases analysed and in 73% of sporadic cases who were *SMARCB1* mutation-negative. Consequently, 27% of sporadic schwannomatosis patients who do not exhibit germline *NF2*, *SMARCB1,* and *LZTR1* mutations, but who do display tumour-specific biallelic *NF2* inactivation, could, in principle, be caused by mutations in hitherto unidentified gene(s). Since Piotrowski et al. (2014) analysed a preselected group of patients and did not include those patients who, although fulfilling the diagnostic criteria for schwannomatosis, lacked biallelic *NF2* inactivation in their tumours, the proportion of unexplained sporadic schwannomatosis cases caused by mutations in other as yet unidentified genes could be even higher. Mutations in these hitherto unidentified genes may also account for familial cases, since Paganini et al. (2015b) investigated six cases with familial schwannomatosis, who were negative for both *SMARCB1* and *LZTR1* mutations. It is possible that some of the unexplained cases are caused by gross rearrangements, multi-exon deletions, or duplications of *SMARCB1* or *LZTR1,* but these lesions are usually much rarer than intragenic mutations and they have not been detected by any of the MLPA or SNP array analyses so far performed (Hadfield et al. 2010a; Smith et al. 2015). Taken together, we conclude that there may well be further schwannomatosis predisposition genes that still remain to be identified.

One possible candidate has been put forward by Zhang et al. (2014) who identified a heterozygous missense mutation, c.622G > C, p.Asp208His, in the *COQ6* gene on chromosome 14q24.3 segregating with the disease in a family with schwannomatosis. The affected family members did not appear to harbour germline mutations in *SMARCB1*, *LZTR1,* or *NF2*; nor were somatic mutations of these genes detected in two schwannomas from two family members. Furthermore, immunohistochemical staining indicated normal protein expression levels of SMARCB1, LZTR1, and NF2 in both schwannomas. The latter finding is unusual, since mosaic SMARCB1 expression has been observed in 93% of schwannomas derived from patients with familial schwannomatosis (Patil et al. 2008). However, Zhang et al. (2014) did not identify biallelic inactivation of *COQ6* gene in schwannomas of this family. Although the deleterious effects of the *COQ6* missense mutation were validated by its lack of complementation in a *coq6*-deficient yeast mutant, there was no evidence for either a dominant-negative effect or a toxic gain of function of the missense *COQ6* variant detected; hence, the mechanism of tumorigenesis in this schwannomatosis family remains unexplained. As opined by Trevisson et al. (2015), further studies are necessary to ascertain whether *COQ6* might play a role in the etiology of schwannomatosis.

*SMARCB1*, *LZTR1,* and *NF2* clearly function as classical tumour suppressor genes, and hence, it is not unreasonable to suppose that other schwannomatosis predisposition genes might also act in an onco-suppressive manner. Although it cannot be excluded that another schwannomatosis predisposition gene is located elsewhere in the genome, it is tempting to speculate that it would be located on chromosome 22, because in schwannomas, this chromosome is frequently affected by LOH. Consequently, the underlying model of tumorigenesis in these unexplained cases could also include three mutational steps similar to the above-mentioned model that accounts for *SMARCB1* and *LZTR1* mutation-positive tumours. Pinto et al. (2014) performed a detailed annotation of the chromosome 22-encoded proteome and identified protein products encoded by 367 genes. Among them were proteins encoded by 22 cancer-associated genes—these may represent good candidates for further schwannomatosis predisposition genes. Finally, a number of other candidate genes may be found among the genes shown by Agnihotri et al. (2016) to be somatically mutated in sporadic schwannomas (see below).

The comprehensive genome-wide analysis of the mutational landscape of somatic mutations in schwannomas of patients with schwannomatosis has not so far been performed but would improve our understanding of the tumorigenic process in schwannomatosis. Such an analysis could also help to identify altered cellular pathways that would reveal the involvement of yet unidentified schwannomatosis predisposition genes. In a recent study, Agnihotri et al. (2016) performed whole-exome sequencing of 13 cranial and 13 spinal schwannomas occurring as sporadic tumours in patients without NF2 or schwannomatosis. No germline mutations or deletions were detected for *NF2*, *LZTR1*, *SMARCB1,* or *SMARCE1*. However, these authors identified 441 somatic single-nucleotide variants located in the exomes of these 26 schwannoma samples, corresponding to 0.16 mutations per coding megabase. This number of mutations is quite low, and comparable to other low-mutation-rate tumours, such as Ewing and rhabdoid sarcomas (Lawrence et al. 2013). Agnihotri et al. (2016) observed somatic *NF2* mutations and/or 22q loss in 96/125 samples (77%). Somatic *LZTR1* mutations were identified in 2/26 schwannomas (8%), but somatic *SMARCB1* mutations were not observed. Recurrent but low-frequency mutations were identified in eight different genes, and Agnihotri et al. validated their findings by targeted sequencing of these genes in an additional 99 sporadic schwannomas. They detected mutations in *DDR1,* encoding a receptor tyrosine kinase activated in lung cancer and other tumour types (Ambrogio et al. 2016), in 14/125 (11%) of the schwannomas. Other recurrently mutated genes were *TSC1* (mutated in 9% of tumours), *CAST* (8%), *ALPK2* (8%), *TSC2* (7%), and *TAB 3* (3%). Furthermore, 29% (37/125) of all schwannomas harboured inactivating mutations in either *ARID1A* or *ARID1B*, which along with *SMARCB1,* encode proteins of the SWI/SNF chromatin-remodeling complex (Nie et al. 2000). Agnihotri et al. (2016) also discovered a somatic recurrent in-frame fusion involving *SH3PXD2A* and *HTRA1*, arising through a balanced translocation on chromosome 10q in 12/25 (10%) of schwannomas analysed by RNA-sequencing. Expression of the *SH3PXD2A*-*HTRA1* fusion in Schwann cells and a schwannoma cell line resulted in increased cell proliferation, invasive growth, and in vivo tumorigenesis. Whether this somatic fusion also contributes to tumorigenesis in the context of schwannomatosis remains to be investigated.

## Conclusion and perspective

Germline mutations in either *SMARCB1* or *LZTR1* predispose to the development of multiple schwannomas in patients with schwannomatosis. In addition, the *NF2* gene is involved in schwannomatosis-associated tumorigenesis, since it is frequently inactivated in schwannomas.

**Fig. 5:**
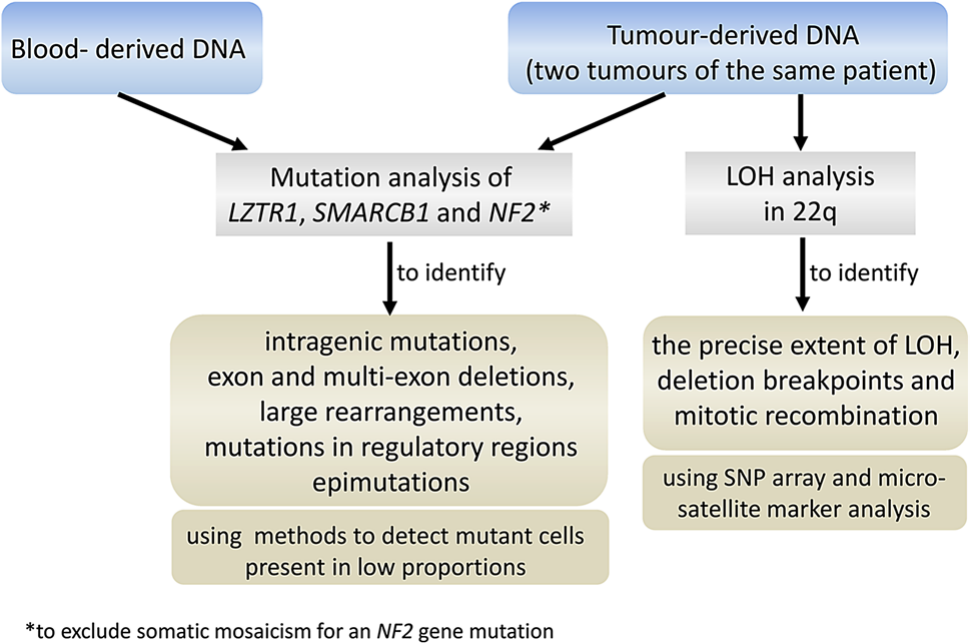
Comprehensive mutation analysis of all three genes, *LZTR1*, *SMARCB1,* and *NF2,* in patients with schwannomatosis should be performed to identify the complete mutational spectra and the number of mutational hits that affect these genes. This comprehensive testing may help to classify the tumours according to their mutation-profile. The mutation analysis should also include methods, such as next-generation sequencing, which are well suited to detect somatic mosaicism with mutant cells present in low proportions. This approach should identify tumour heterogeneity and help to distinguish between mosaic NF2 and schwannomatosis, since some NF2 patients with somatic mosaicism for an *NF2* gene mutation fulfil the diagnostic criteria for schwannomatosis (Plotkin et al. 2013)

However, determining the complete mutational spectrum of all three genes, *LZTR1*, *SMARCB1,* and *NF2,* by comprehensive mutation testing as suggested in Fig. 5, has not so far been attempted. Such comprehensive testing would help to classify schwannomas in terms of the number of mutational hits and the biallelic or mono-allelic inactivation of these tumour suppressor genes (TSGs). A genetic classification of schwannomas might correlate with tumour growth patterns, physical location, and response to therapy.

From the data so far available, it is already clear that at least two and sometimes three TSGs are inactivated in schwannomas from patients with schwannomatosis, an event which is frequently mediated by LOH, including large portions of 22q. The possible involvement of further TSGs in predisposition to schwannomatosis would serve to render the mutational model even more complex.

Recurrent cancer-associated deletions involving several linked TSGs have also been observed on chromosomes 7q, 8p, and 9p in different types of tumour (Krimpenfort et al. 2007; Asou et al. 2009; Solimini et al. 2012; Xue et al. 2012; Kotini et al. 2015). The concomitant loss of several TSGs as a consequence of a single mutational event, such as a gross chromosomal deletion, may disclose an interactive or cooperative effect in the sense that the biallelic inactivation and haploinsufficiency of several TSGs collectively promote tumorigenesis of the affected cells, since several signalling pathways and other cellular processes are concurrently disturbed. Schwannomatosis serves as a paradigm for such cooperative tumorigenic effects mediated by the concomitant loss of several linked TSGs.

## Electronic supplementary material

Below is the link to the electronic supplementary material.

Supplementary material 1 (DOC 215 kb)
