# Supplementary material for: The molecular pathogenesis of schwannomatosis, a paradigm for the co-involvement of multiple tumour suppressor genes in tumorigenesis
Source: Hum Genet. 2016 Dec 5;136(2):129–48. doi: 10.1007/s00439-016-1753-8 (PMC5258795; doi:10.1007/s00439-016-1753-8)
Supplement: Supplementary file 1 — Supplementary material 1 (DOC 215 kb) [file 439_2016_1753_MOESM1_ESM.doc]

# Supplementary Table S1: *SMARCB1* mutations identified in patients with schwannomatosis. *SMARCB1* includes nine exons. The positions of the mutations are indicated according to the reference sequence of *SMARCB1* (NM_003073.3)

| **Genomic mutation** | **Location of mutation** | **Type of mutation and actual/predicted consequences as far as they have been investigated** | **Acutal/ predicted protein alteration (RNA alteration)** | **Reference** |
| --- | --- | --- | --- | --- |
| c.30delC | Exon 1 | The frameshift mutation is predicted to cause a premature termination codon. The mutant transcripts are however stable and translation is reinitiated at position 27 of the amino acid sequence of SMARCB1 giving rise to N-terminally truncated proteins. | p.Phe10Leufs*6 | Rousseau et al. (2011); Hulsebos et al. (2014a) |
| c.34C>T | Exon 1 | Nonsense mutation; the mutant transcripts are however stable and translation is reinitiated at position 27 of the amino acid sequence of SMARCB1 giving rise to N-terminally truncated proteins. | p.Gln12* | Hulsebos et al. (2007); Rousseau et al. (2011); Smith et al. (2012b); Hulsebos et al. (2014a) |
| c.38delA | Exon 1 | The frameshift mutation is predicted to cause a premature termination codon. The mutant transcripts are however stable and translation is reinitiated at position 27 of the amino acid sequence of SMARCB1 giving rise to N-terminally truncated proteins. | p.Lys13Serfs*3 | Smith et al. (2014); Hulsebos et al. (2014a) |
| c.40C>T | Exon 1 | Missense | p.Pro14Ser | Smith et al. (2014) |
| c.41C>T | Exon 1 | Missense | p.Pro14His | Hadfield et al. (2008); Smith et al. (2012b) |
| c.41C>A | Exon 1 | Missense mutation; the mutant mRNA has been confirmed to be stable. SMARCB1 expression vectors with this mutation were functional in a cyclin D1 transcription suppression assay. | p.Pro14His  (r.41C>A) | Boyd et al. (2008); Smith et al. (2012c) |
| c.46A>T | Exon 1 | Nonsense mutation; the mutant transcripts harbouring this mutation are not degraded and translation is reinitiated at position 27 of the amino acid sequence of SMARCB1 giving rise to N-terminally truncated proteins. | p.Lys16* | Hadfield et al. (2008); Hulsebos et al. (2014a) |
| c.86_91del | Exon 1 | In-frame deletion | p.Gly29_Ser30del | Hadfield et al. (2008); Smith et al. (2014) |
| c.92A>T | Exon 1 | Missense mutation | p.Glu31Val | Bacci et al. (2010); Smith et al. (2012b) |
| c.143C>T | Exon 2 | Missense mutation | p.Pro48Leu | Christiaans et al. (2010); van den Munckhof et al. (2012) |
| c.158G>T | Exon 2 | Missense mutation; the mutant mRNA has been confirmed to be stable. SMARCB1 expression vectors with this mutation were functional in a cyclin D1 transcription suppression assay. | p.Arg53Leu  (r.158G>U) | Boyd et al. (2008); Smith et al. (2012c) |
| c.203_216delinsTACC | Exon 2 | 14-bp deletion associated with a 4-bp insertion causing a frameshift that is predicted to lead to the introduction of a premature termination codon. | p.His68Leufs*14 | Sestini et al. (2008) |
| c.207_208dupTA | Exon 2L | Splice site mutation located in the alternative spliced sequences of exon 2, affecting only the long exon 2 (2L) coding sequence of the isoform 1 transcript. However, the alteration was located at the +4 position in intron 2S. The mutation is predicted to interfere with the splicing of SMARCB1 isoform 1 but not isoform 2. |  | Melean et al. (2012) |
| c.233-1G>A | Exon 3 | Splicing alteration; the mutation disrupts the intronic-acceptor splice site involved in the pre-mRNA splicing of introns 2S and 2L. |  | Smith et al. (2012b); Melean et al. (2012) |
| c.233-2_237 delagATCAC | Exon 3 | Splicing alteration; the 7-bp deletion at the start of exon 3 removes the splice acceptor site. It is predicted to create an alternative splice site resulting in insertion of intronic sequence which introduces a premature termination signal at codon 71. |  | Hadfield et al. (2008) |
| c.362+1G>A | Intron 3 | Splice site mutation causing an in-frame deletion of the last 45-bp of exon 3. | (r.318_362del) | Boyd et al. (2008); Smith et al. (2012c) |
| c.364G>T | Exon 4 | The mutation causes the skipping of exon 4 without changing the reading frame. SMARCB1 expression vectors with this mutation were functional in a cyclin D1 transcription suppression assay. | (r.363_500del) | Boyd et al. (2008); Smith et al. (2012c) |
| c.392G>A | Exon 4 | Nonsense mutation | p.Trp131* | Wu et al. (2015) |
| c.500G>A | Exon 4 | Splicing alteration; the mutation affects the last base of exon 4, and alters the splicing which results in the in-frame loss of the last 111-bp of exon 4 (c.390_500del). | (r.390_500del) | Boyd et al. (2008); Smith et al. (2012c) |
| c.500+5G>T | Intron 4 | Splicing alteration which results in the in-frame loss of the last 111-bp of exon 4 (c.390_500del). | (r.390_500del) | Boyd et al. (2008); Smith et al. (2012c) |
| c.[500+5G>T,505G>T] | Intron 4 | Splice alteration causing a frameshift predicted to lead to the introduction of a premature termination codon | p.[Arg121_Cys167delinsSer,Gly169*] | Rousseau et al. (2011) |
| c.501-23T>G | Intron 4 | Splicing alteration |  | Rousseau et al. (2011) |
| c.629-5T>G | Intron 5 | Splicing alteration. This mutation appears to disrupt the exon 6 splice acceptor sequence leading to the insertion of 145-bp of the 3′ end of intron 5 using a cryptic splice acceptor (ag/TTT). This alteration is predicted to lead to the introduction of a premature stop codon within the intronic sequence. | (r.500ins501-145_501-1) | Boyd et al. (2008); Smith et al. (2012c) |
| c.795+1G>T | Intron 6 | Splice alteration. The mutation creates two alternative splice variants: one leads to the in-frame inclusion of the first 45-bp of intron 6 whereas the second (which was much less abundant) involved the skipping of exon 6 causing a frame-shift predicted to lead to a premature stop codon. | (r.795_796ins795+1_795+45ins, r.628_795del) | Boyd et al. (2008); Smith et al. (2012c) |
| c.832C>T | Exon 7 | Nonsense mutation | p.Gln278* | Rousseau et al. (2011) |
| c.864C>G | Exon 7 | Missense mutation | p.Asn288Lys | Hadfield et al. (2008) |
| c.971_978delinsTGCTACCT | Exon 7 | In-frame deletion and insertion | p.Lys324_Tyr326  delinsIleLeuPro | Rousseau et al. (2011) |
| c.796-2246_9861 +5250dup7686 | Exon 7 | Duplication of exon 7 causing a frameshift and the introduction of a premature termination codon | p.Glu330* | Hulsebos et al. (2016)**^a^** |
| c.1000_1001insG | Exon 8 | Frameshift mutation | p.Pro334Argfs*27 | Smith et al. (2014) |
| c.1021delC | Exon 8 | Frameshift mutation | p.Arg341Glyfs*16 | Smith et al. (2012b) |
| c.1032-12C>G | Intron 8 | Splicing alteration |  | Hadfield et al. (2008) |
| c.1106A>T | Exon 8 | Missense mutation | p.Asp369Val | Hadfield et al. (2008) |
| c.1118G>A | Exon 8 | The mutation represents a splice alteration involving the last base of exon 8 and disrupting the donor splice site. The derivative RNA is predicted to encode an elongated protein with a new translation stop codon after 48 amino acids. |  | Paganini et al. (2015a)**^b^** |
| c.1118+1G>A | Intron 8 | The mutation alters the donor splice site sequence of intron 8. This causes the skipping of exon 8 which is predicted to lead to an in-frame deletion of 132 nucleotides in the transcript. |  | Hulsebos et al. (2010) |
| c.*82C>T  [c.1240C>T] | 3'UTR | Transcripts harbouring the mutation did not produce splice variants but instead exhibited reduced expression as compared with the wild-type mRNA. |  | Boyd et al. (2008); Smith et al. (2012b); Smith et al. (2012c); Asai et al. (2015) |

**^a^** Hulsebos et al. (2016) identified this mutation in a patient with schwannomatosis and a type 1 papillary renal cell carcinoma (pRCC1).

**^b^** The patient reported by Paganini et al. (2015a) had schwannomatosis and a uterine leiomyosarcoma

# Supplementary Table S2: Frequency of *SMARCB1* mutations in familial and sporadic schwannomatosis

| **Number of *SMARCB1* mutation carriers/ number of schwannomatosis patients screened [proportion]** | | **References** |
| --- | --- | --- |
| **familial** | **sporadic** |
| 13/19 [68.4%] | - | Boyd et al. (2008) |
| 5/15 [33.3%] | 2/28 [7.1%] | Hadfield et al. (2008)**^a^** |
| 0/2 | 1/19 [5.3%] | Sestini et al. (2008) |
| 0/2 | 5/54 [9.3%] | Rousseau et al. (2011) |
| 4/11 [36.4%] | 8/89 [8.9%] | Smith et al. (2012b) |
| 3/4 [75%] | 6/25 [24%] | Smith et al. (2014) |
| Σ 25/53 [47%] | Σ 22/215 [10.2%] |  |
| Σ 26/54 [48%]**^a^** | Σ 21/214 [9.8%]**^a^** |  |

**^a^** The numbers have been corrected according to Smith et al. (2014) who reclassified one ‘sporadic’ case from their earlier study (Hadfield et al. 2008) as familial.

# Supplementary Table S3: *SMARCB1* germline mutations identified in families with individuals exhibiting multiple schwannomas and/or meningiomas

| ***SMACRB1* germline mutation** | **Affected family members** | **Reference** |
| --- | --- | --- |
| Exon 1 c.92A>T (p.Glu31Val)**^a^** | Four family members were affected in two generations. One family member had a schwannoma but further investigations were not performed. Two patients exhibited multiple schwannomas and meningiomas; one family member presented with only meningiomas but spinal MRI was not performed. | Bacci et al. (2010) |
| Exon 2 c.143C>T; p.Pro48Leu | Three-generation family with 18 mutation carriers; 11 family members were clinically investigated and found to exhibit multiple meningiomas and schwannomas. | Christiaans et al. (2011) van den Munckhof et al. (2012) |
| c.233-1G>A splice-site mutation in intron 2, causing a frameshift and a premature stop codon at residue 71 (p.Asp69Glyfs*2)**^b^** | Four family members were affected in three generations. Two members of this family exhibited schwannomas; one member had schwannomas and an intracranial meningioma whereas another member had two intracranial meningiomas and an intraorbital schwannoma at birth. | Melean et al. (2012) |

**^a^** This missense mutation was also identified in a sporadic patient with a meningioma (Smith et al. 2012b).

**^b^** This splice-site mutation was also detected in a patient with schwannomatosis but without meningiomas (Smith et al. 2012b).

# Supplementary Table S4: Recurrent mutations identified in *SMARCB1*-associated schwannomatosis

| **Total number of germline *SMARCB1* mutations identified** | **Number of patients with *SMARCB1* mutation / encoded protein alteration** | | | | **Reference** |
| --- | --- | --- | --- | --- | --- |
|  | c.*82C>T  (3’ UTR)**^a^** | c.41C>A /  p.(Pro14His) | c.34C>T /  p.(Gln12*) | c.86_91del / p.(Gly29_Ser30del) |  |
|  |  |  | 1 |  | Hulsebos et al. (2007) |
| 13 | 4 | 1 |  |  | Boyd et al. (2008) |
| 7 |  | 1 |  | 2 | Hadfield et al. (2008) |
| 5 | - | - | 1 |  | Rousseau et al. (2011) |
| 3 | - | - |  |  | Sestini et al. (2008) |
| 12 | 5 | 1 | 1 |  | Smith et al. (2012b) |
| 9 | 4 | - |  | 1 | Smith et al. (2014) |
| 1 | 1 |  |  |  | Asai et al. (2015) |
| Σ=50 | Σ=14 [28%] | Σ=3 [6%] | Σ=3 [6%] | Σ=3 [6%] |  |

**^a^** Transcripts harbouring the mutation did not produce splice variants but showed reduced expression as compared with the wild-type mRNA (Smith et al. 2012c).]

# Supplementary Table S5: Families with germline *SMARCB1* mutations and schwannomatosis and/or rhabdoid tumour (RT)

| **Number of affected family members with schwannomas and/or rhabdoid tumour (RT)** | ***SMARCB1* germline mutation** | ***SMARCB1* somatic (tumour-specific) mutation** | **SMARCB1 expression in the tumour** | **Reference** |
| --- | --- | --- | --- | --- |
| Four generations were affected by either schwannomatosis or RT. Five clinically affected family members were confirmed to be *SMARCB1* mutation carriers. Two family members were presumed to be clinically affected whereas two family members harbouring the mutation were asymptomatic. Schwannomatosis was clinically confirmed in two mutation carriers. Two family members exhibited ‘skin lumps’ suggestive of schwannomas whilst two members carrying the mutation died from RT. | Direct duplication of 2,631-bp including exon 6 which resulted in a frameshift and protein truncation (p.Leu266fs). | Deletion due to 22q loss in one RT; intragenic mutation c.578_585dup8 in the RT of the second child; 22q LOH in one schwannoma. | One schwannoma was investigated; this was designated as an epithelioid variant and exhibited complete loss of SMARCB1 expression. | Swensen et al. (2009) |
| The female propositus harboured a *SMARCB1* germline mutation and was affected by RT. Her father and paternal grandmother were also carriers of the mutation and had schwannomatosis but not RT. | c.472C>T, exon 4**^a^** | Deletion of the second *SMARCB1* allele in RT. | Complete absence of SMARCB1 protein expression in RT | Eaton et al. (2011)  Smith et al. (2012c) |
| Female propositus harboured a *de novo* *SMARCB1* germline mutation and multiple neuroblastoma-like schwannomas arising from the peroneal and ulnar nerves. One of these schwannomas transformed to malignancy and was diagnosed as an epithelioid malignant peripheral nerve sheath tumour (MPNST) with rhabdoid features. All three of the propositus’ children harboured the mutation, two of whom had RT, whilst one child was asymptomatic. | Frameshift mutation c.245_246insAT, exon 3 | Loss of the second *SMARCB1* allele in the RT of the son and in the MPNST of the mother. | Complete loss of SMARCB1 expression in the MPNST | Carter et al. (2012) |
| Two sisters exhibited RT, their father and paternal grandmother had schwannomatosis. | Not investigated | Not investigated | Not investigated | Sredni and Tomita, 2015 |

**^a^**: The consequences of this mutation were further explored by Smith et al. (2012c). Three alternately spliced cDNA amplicons were derived from the RNA of a lymphoblast cell line established from blood cells of one of the family members. The PCR amplification of exons 3–6 detected two different deletions: the complete loss of exon 4 (c.363_500del) and a partial exon 4 deletion (c.390_500del). Amplicons for exons 5-9 indicated a complete deletion of exon 7 predicted to generate a premature stop codon which would lead to nonsense-mediated decay.

# Supplementary Table S6: Number of schwannomas with mono-allelic *NF2* inactivation in patients with schwannomatosis

| Number of schwannomas with mono-allelic *NF2* inactivation/ total number of schwannomas analysed from patients with *SMARCB1* germline mutations | Reference |
| --- | --- |
| 3/13 | Boyd et al. (2008) |
| 1/3 | Sestini et al. (2008) |
| 0/8 | Hadfield et al. (2008) |
| 2/8 | Hadfield et al. (2010) |
| Σ= 6/32 (19%) |  |

# Supplementary Table S7: Frequency of germline *LZTR1* mutations in patients with schwannomatosis without germline *NF2* or *SMARCB1* mutations

| **Total number of unrelated patients screened** | **Number of *LZTR1* mutation carriers/ number of patients screened [proportion]** | | References |
| --- | --- | --- | --- |
|  | **familial** | **sporadic** |  |
| 20 | 6/6 [100%] | 8/11 [73%] | Piotrowski et al. (2014) |
| 23**^a^** | - | 5/22 [23%] | Hutter et al. (2014) |
| 72 | 3/8 [38%] | 19/64 [30%] | Paganini et al. (2014) |
| 65 | 6/16 [38%] | 11/49 [22%] | Smith et al. (2015) |

**^a^**: One patient did not exhibit a *SMARCB1* mutation in blood lymphocytes but an identical *NF2* gene mutation was observed in two different schwannomas indicative of mosaic NF2.

# Supplementary Table S8: *LZTR1* mutations identified in patients with schwannomatosis. Mutations highlighted in grey were detected in both affected and non-affected family members. The position of the mutations is according to the reference sequence of *LZTR1* (NM_006767.3).

| **Genomic mutation** | **Location of mutation** | **Type of mutation^a^** | **Protein alteration** | **Re-ference** | **Unaffected mutation carriers^b^** |
| --- | --- | --- | --- | --- | --- |
| c.27delG | Exon 1 | Frameshift/ truncating | p.Gln10Argfs*15 | 1,5 | **+** |
| c.212A>G | Exon 2 | Missense/ deleterious | p.His71Arg | 2 |  |
| c.238dupA | Exon 2 | Frameshift/ truncating | p.Lys89Cysfs*15 | 1 |  |
| c.243T>G | Exon 2 | Nonsense | p.Tyr81* | 2 | **+** |
| c.264-13G>A | Intron 2 | Splice site/ Frameshift/ truncating | p.Lys89Cysfs*16 | 1 |  |
| c.321-2delA | Intron 3 | Splice site/ skipping of exon 4/ out-of-frame transcript |  | 4 |  |
| c.347_348insC | Exon 4 | Frameshift | p.A116fs | 4 |  |
| c.352dupC | Exon 4 | Frameshift/ truncating | p.Arg118Profs*28 | 2 | **+** |
| c.365C>T | Exon 4 | Missense/ deleterious | p.Ser122Leu | 1 |  |
| c.373_375delGTC | Exon 4 | Inframe deletion | p.Val125del | 2 |  |
| c.401-2A>G | Exon 5 | Splice site | unknown | 3 |  |
| c.509G>A | Exon 5 | Missense/ deleterious | p.Arg170Gln | 3 |  |
| c.513delG | Exon 5 | Frameshift/ truncating | p.Leu171Phefs*29 | 2 |  |
| c.555_556dupCA | Exon 6 | Frameshift/ truncating | p.Lys186Thrfs*15 | 2 |  |
| c.560T>G | Exon 6 | Missense/ deleterious | p.Leu187Arg | 2 |  |
| c.570delT | Exon 6 | Frameshift | p.Phe190Leufs*10 | 3,5 |  |
| c.594-3C>G | Intron 6 | Splice site/ frameshift/ truncating | p.Leu199Trpfs*34 | 1 | **+** |
| c.605T>G | Exon 7 | Missense/ deleterious | p.Met202Arg | 3 |  |
| c.628C>T | Exon 7 | Nonsense | p.Arg210* | 2 |  |
| c.791+1G>A | Exon 8 | Splice site/ predicted skipping of exon 8/ out-of-frame transcript |  | 2 |  |
| c.842delC | Exon 9 | Frameshift/ truncating | p.Pro281Argfs*70 | 3 |  |
| c.850C>T | Exon 9 | Missense/ deleterious | p.Arg284Cys | 2 |  |
| c.856G>A | Exon 9 | Missense/ deleterious | p.Gly286Arg | 3 |  |
| c.964G>T | Exon 9 | Nonsense | p.Glu322* | 3 |  |
| c.1018C>T | Exon10 | Nonsense | p.Arg340* | 2 |  |
| c.1175C>T | Exon 11 | Missense/ deleterious | p.Ala392Val | 3 |  |
| c.1199T>G | Exon 11 | Missense/ deleterious | p.Met400Arg | 2 |  |
| c.1210G>A | Exon 11 | Missense/ deleterious | p.Gly404Arg | 1,5 |  |
| c.1312G>T | Exon 12 | Nonsense | p.Glu438* | 4 |  |
| c.1353+1G>A | Exon 12 | Splice site | unknown | 3 |  |
| c.1367T>G | Exon 13 | Missense/ deleterious | p.Val456Gly | 1 |  |
| c.1373dupG | Exon 13 | Frameshift/ truncating | p.His459Profs*210 | 2 |  |
| c.1394C>A | Exon 13 | Missense/ deleterious | p.Ala465Glu | 2 |  |
| c.1397G>A | Exon 13 | Missense/ deleterious | p.Arg466Gln | 1 |  |
| c.1449+1G>A | Intron 12 | Splice site/ in frame skipping of exon 13 | p.Glu453_Lys484del | 1 |  |
| c.1480_1481insAG | Exon 14 | Frameshift | p.Arg494fs | 4 |  |
| c.1483dupG | Exon 14 | Frameshift/ truncating | p.Glu495Glyfs*174 | 3 |  |
| c.1486delG | Exon 14 | Frameshift/ truncating | p.Ala496Profs*60 | 2 |  |
| c.1559C>T | Exon 14 | Missense/ deleterious | p.Pro520Leu | 1 |  |
| c.1583T>G | Exon 14 | Missense/ deleterious | p.Leu528Arg | 3 |  |
| c.1602delA | Exon 14 | Frameshift/ truncating | p.Lys534Asnfs*22 | 2 | **+** |
| c.1751dupA | Exon 15 | Frameshift/ truncating | p.Ser585Glufs*84 | 1 |  |
| c.1779delA | Exon 15 | Frameshift/ truncating | p.Gln593Hisfs*7 | 2 |  |
| c.1785+2delT | Exon 15 | Splice site/ unknown |  | 5 |  |
| c.1807delG | Exon 15 | Frameshift/ truncating | p.Val603* | 2 | **+^c^** |
| c.1893delG | Exon 16 | Frameshift/ truncating | p.Lys632Serfs*20 | 3 |  |
| c.1961A>G | Exon 17 | Missense/ deleterious | p.Asp654Gly | 3 |  |
| c.2002G>T | Exon 17 | Missense/ deleterious | p.Asp668Tyr | 3 |  |
| c.2062C>T | Exon 17 | Missense/ deleterious | p.Arg688Cys | 3 |  |
| c.2062C>T | Exon 17 | Missense/ deleterious | p.Arg688Cys | 1 | **+** |
| c.2062C>T | Exon 17 | Missense/ deleterious | p.Arg688Cys | 1 |  |
| c.2089C>T | Exon 18 | Missense/ deleterious | p.Arg697Trp | 2 |  |
| c.2220-16_2220-14delCTT | Intron 18 | Splice site? | unknown | 1 |  |
| c.2247C>A | Exon 19 | Nonsense | p.Tyr749* | 4 |  |
| c.2278T>C | Exon 19 | Missense/ deleterious | p.Cys760Arg | 2 | **+** |
| c.2284C>T | Exon 19 | Nonsense | p.Gln762* | 3 | **+** |
| c.2348_2351delCGCA | Exon 20 | Frameshift/ truncating | p.Thr783Argfs*5 | 1 | **+** |
| c.2438G>T | Exon 21 | Missense/ deleterious | p.Ser813Ile | 1 |  |
| c.2487dupA | Exon 21 | Frameshift/ truncating | p.Asp830Argfs*21 | 2 |  |

**^a^** These missense mutations were predicted to be deleterious by MutationTaster, PolyPhen 2 and SIFT

**^b^** Mutations highlighted in grey and marked with a "+" in this column appear to be characterized by variable penetrance because they were observed in clinically unaffected as well as affected family members.

**^c^** It is unclear whether this *LZTR1* mutation is the pathogenic mutation in this family since it was not detected in the affected brother of the propositus. The father of the propositus was not affected but did harbour the mutation. The mutant *LZTR1* allele was retained in two tumours of the propositus.

1: Piotrowski et al. (2014)

2: Paganini et al. (2015b)

3: Smith et al. (2015a)

4: Hutter et al. (2014)

5: Smith et al. (2016)
